# Genome-based assessment of antimicrobial resistance of *Escherichia coli* recovered from diseased swine in eastern China for a 12-year period

**DOI:** 10.1128/mbio.00651-25

**Published:** 2025-04-17

**Authors:** Junxing Li, Jiang Chang, Jiangang Ma, Wei Zhou, Yue Yang, Jing Wu, Chunjiu Guan, Xiufang Yuan, Lihua Xu, Bin Yu, Fei Su, Shiyi Ye, Yijie Chen, Guoping Zhao, Biao Tang

**Affiliations:** 1 Institute of Animal Husbandry and Veterinary Science, Zhejiang Academy of Agricultural Sciences 74561 , Hangzhou, Zhejiang, China; 2 Key Laboratory of Systems Health Science of Zhejiang Province, School of Life Science, Hangzhou Institute for Advanced Study, University of the Chinese Academy of Sciences 638898 , Hangzhou, Zhejiang, China; 3 State Key Laboratory for Managing Biotic and Chemical Threats to the Quality and Safety of Agro-Products, Institute of Agro-product Safety and Nutrition, Zhejiang Academy of Agricultural Sciences 74561, Hangzhou, Zhejiang, China; 4 Xianghu Laboratory 665999 , Hangzhou, Zhejiang, China; 5 Zhejiang Provincial Center for Animal Disease Prevention and Control, Hangzhou, China; 6 National Genomics Data Center & Bio-Med Big Data Center, CAS Key Laboratory of Computational Biology, Shanghai Institute of Nutrition and Health, Chinese Academy of Sciences 26443, Shanghai, China; McMaster University Department of Biochemistry & Biomedical Sciences, Hamilton, Canada

**Keywords:** *Escherichia coli*, swine, antimicrobial resistance, *mcr*, *tet*(X4)

## Abstract

The global rise of antimicrobial resistance (AMR), driven by antibiotic use in healthcare and agriculture, poses a major public health threat. While AMR in clinical settings is well studied, there is a gap in understanding the resistance profiles of *Escherichia coli* from diseased livestock, particularly regarding zoonotic transmission. This study analyzes 114 *E. coli* isolates from diseased swine over 12 years, revealing that 99.12% were multidrug-resistant. Resistance was highest for ampicillin and amoxicillin/clavulanic acid (100%), followed by ciprofloxacin (96.49%) and tetracycline (94.74%). Furthermore, 21.05% of isolates were resistant to colistin, and 1.75% to tigecycline. A total of 76 antimicrobial resistance genes (ARGs) were identified, with *mcr-1* found in 18.42%, *mcr-3* in 4.39%, and *tet*(X4) in 1.75%. Significant co-occurrence of ARGs and plasmids suggests potential for co-selective dissemination. This study is the first to report enterotoxigenic *E. coli* (ETEC) strains carrying both *mcr-1* and *mcr-3* genes. After the 2017 colistin ban in China, *mcr-1* detection rates significantly decreased, while florfenicol resistance rates increased in 2018–2021 (94.29%) compared to 2010–2017 (79.55%). This work provides valuable insights into the AMR profiles of *E. coli* from diseased swine and highlights trends that can inform strategies for monitoring and controlling public health risks associated with zoonotic *E. coli* transmission.

**IMPORTANCE:**

This study highlights the critical role of diseased and deceased swine in the spread of antimicrobial resistance (AMR), providing new insights into the transmission of resistance genes in zoonotic contexts. By analyzing *E. coli* from diseased swine, we identify key resistance genes such as *mcr-1*, *mcr-3*, and *tet*(X4), which pose significant public health risks, especially regarding last-resort antibiotics like colistin. Moreover, the study identifies novel transmission patterns of *mcr* genes, including ETEC strains carrying the *mcr-3* gene and strains harboring both *mcr-1* and *mcr-3* genes. The role of plasmids in horizontal gene transfer is also revealed, facilitating rapid AMR spread across species. The long-term persistence of resistant strains highlights the challenges in controlling AMR in livestock. These findings underscore the need for enhanced surveillance and a One Health approach to mitigate AMR risks across animal, human, and environmental health.

## INTRODUCTION

The utilization of antimicrobial agents, primarily used for prophylaxis and growth promotion in livestock farming as well as for treating infections in clinical settings, has significantly contributed to human civilization in recent decades (1). However, the inappropriate use during this period has driven the emergence and global dissemination of antimicrobial-resistant pathogens carrying antibiotic resistance genes (ARGs) (2). This evolutionary pressure has triggered a concerning trajectory where some antimicrobial agents have seen marked reductions in efficacy, with several becoming obsolete due to resistant strains (3). Particularly alarming is the emergence of critical acquired ARGs, including the carbapenemase gene *bla*_NDM_ (4), extended-spectrum β-lactamase gene *bla*_KPC_ (5), colistin resistance gene *mcr-1* (6), and tigecycline resistance gene *tet*(X4/X5) (7, 8), which confer resistance to last-resort antimicrobials. These developments render clinically available treatments potentially ineffective against associated infections. Compounding this crisis, the dearth of novel antibiotics in development pipelines suggests that the threat posed by pan-resistant pathogens will persist indefinitely (9).

*Escherichia coli* is a zoonotic opportunistic pathogen of global concern, capable of bidirectional transmission between humans and animals (2). While many *E. coli* strains are commensal, pathogenic variants can induce clinical manifestations ranging from self-limiting diarrhea to life-threatening hemorrhagic colitis and systemic infections (10). Of particular concern is its role as a mobile genetic reservoir, persistently acquiring and disseminating antimicrobial resistance determinants across ecological boundaries (11). This dual threat is amplified by the species’ genomic plasticity, enabling simultaneous carriage of virulence factors and resistance cassettes on integrative conjugative elements (12). As a pivotal ARG disseminator, *E. coli* carries high-risk resistance loci such as *bla*_NDM-5_, *mcr-1*, and *tet*(X4/X5), serving as critical vectors for intersectoral ARG flux at the human-animal-environment interface (13). The convergence of hypervirulence and multidrug resistance in *E. coli* lineages elevate zoonotic risks, threatening agricultural productivity through livestock pandemics while complicating clinical infection management (14).

The emergence of whole-genome sequencing (WGS) technology has facilitated the identification of comprehensive genetic determinants that underlie major mechanisms of antimicrobial resistance (AMR) and virulence with ease (15). Such information can be exchanged among nations for comparative analysis, thus strengthening global control measures against infectious diseases. WGS plays a pivotal role in characterizing antibiotic-resistant pathogens as well as detecting their associated ARGs (16).

Previous research has identified certain characteristics of *E. coli* as a vector for AMR dissemination under non-selective conditions (6). However, there is a scarcity of studies focusing on the AMR of *E. coli* strains isolated from diseased or dead animals. These strains may exhibit higher pathogenicity and present a greater potential threat to livestock, poultry, and human health. In this study, we conducted a comprehensive analysis of *E. coli* strains from diseased swine or dead swine over a 12-year period to elucidate their epidemic characteristics. These isolates were then subjected to genomic analysis using WGS technology, providing a systematic interpretation of genomic information on AMR and virulence traits, with the hope of conveying essential insights for preventing and treating animal and clinical diseases associated with *E. coli*.

## RESULTS

### The prevalence of *E. coli* in this study

**Fig 1:**
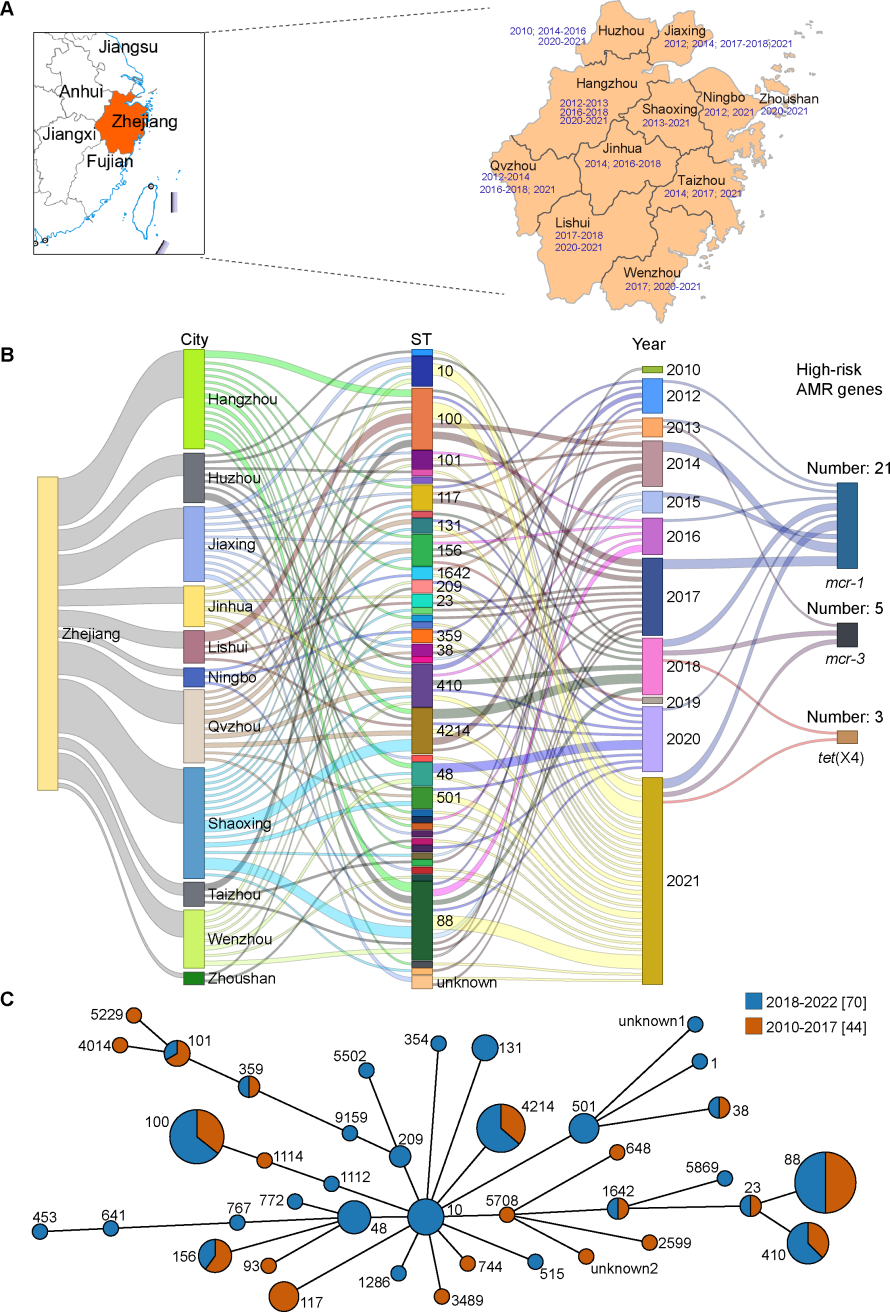
The prevalence characteristic of 114 *E. coli* isolates. (A) Geographical distribution of the sampling areas in Zhejiang Province, China. The isolation time of samples in each city was marked in blue. (B) Sankey diagram combining the cities, STs, years, and high-risk ARGs based on 114 E. coli isolates. The diameter of the line is proportional to the number of isolates. (C) A minimum spanning tree of *E. coli* isolates based on MLST. Each node represents a single ST. The size of the nodes is proportional to the number of isolates. The length of branches between each node is proportional to the number of different alleles that differ between two linked nodes.

Between July 2010 and October 2021, a total of 114 *E. coli* strains isolated from swine that suffered from or died of diarrhea, splenomegaly, hepatomegaly, or other diseases were collected from 82 farms across 11 cities (Huzhou, Ningbo, Jiaxing, Quzhou, Hangzhou, Shaoxing, Taizhou, Jinhua, Lishui, Wenzhou, and Zhoushan) in Zhejiang Province (Fig. 1A; Table S1).

As shown in Fig. 1B and C; Table S1, a total of 39 different ST patterns (including two new STs) were identified among 114 *E. coli* isolates, ST88 was the most commonly represented by 18 (15.79%, 18/114) isolates, followed by ST100 (12.28%, 14/114) and ST4214 (9.65%, 11/114). There were at least two ST patterns in each city, mostly found in Shaoxing (14 ST patterns), followed by Hangzhou (13 ST patterns).

Based on the ST patterns and isolation times of *E. coli* isolates analyzed in this study, we investigated the potential relationship between these two factors. The results of the comparative analysis revealed that multiple ST patterns typically coexist within a single year. This finding suggests that the sources and transmission routes of *E. coli* infections are likely diverse, rather than stemming from a single source or clonal group. Consequently, it is improbable that these infections result from a centralized outbreak driven by a single transmission chain (Fig. 1B). Interestingly, it was also observed that some ST patterns were only found in 2010–2017 such as ST117 and ST93, while some ST patterns were only found in 2018–2021 such as ST48 and ST10 (Fig. 1C). At the same time, some mostly identified ST patterns showed long-time prevalence. For example, ST88 was identified in 9 years (2010, 2012, 2014, 2015, 2016, 2017, 2018, 2020, and 2021) (Fig. 1B).

**Fig 2:**
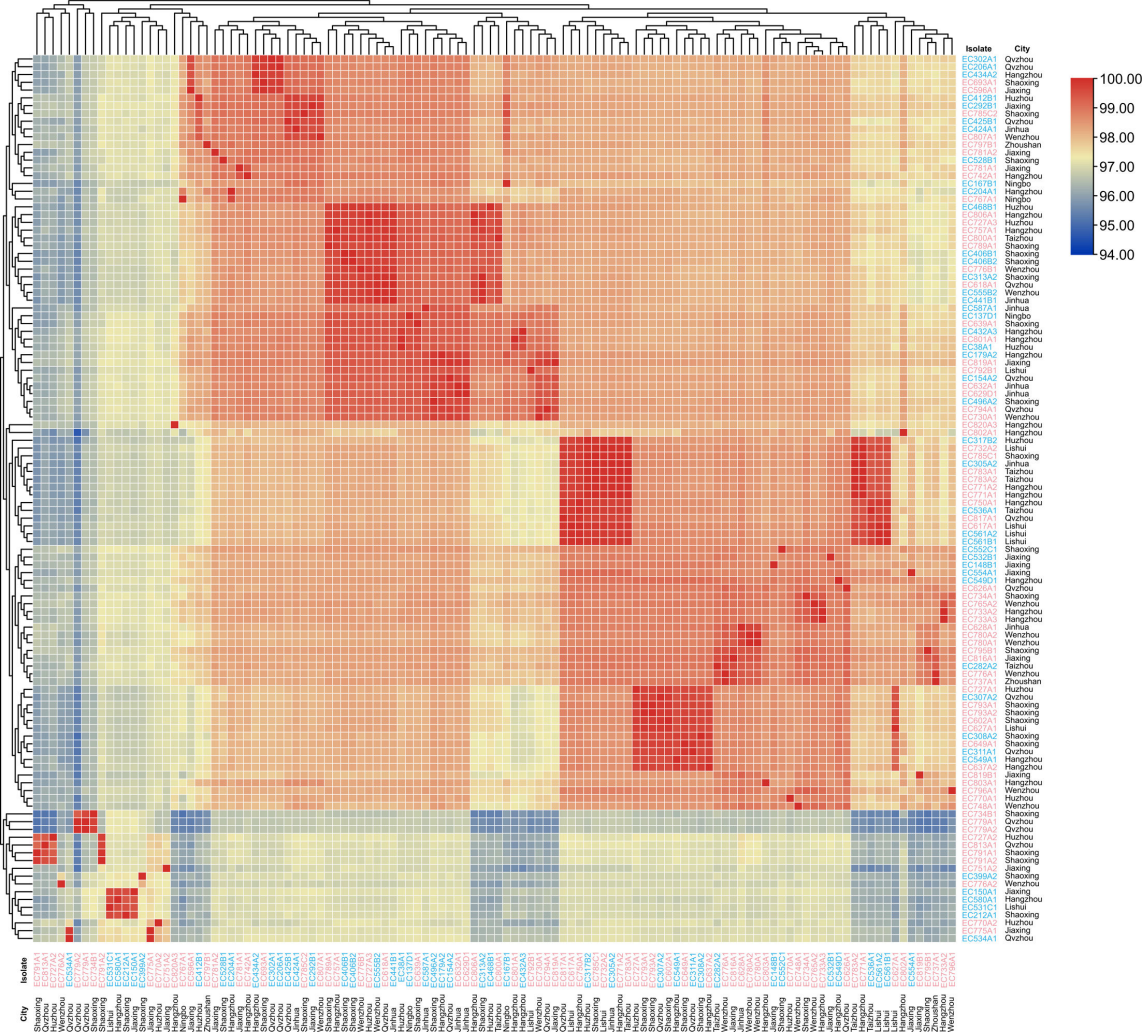
The ANIs among 114 *E. coli* isolates. The name of *E. coli* strains that were isolated in 2010–2017 was marked in blue, and the name of *E. coli* strains that were isolated in 2018–2021 was marked in pink.

All isolates were detected as *E. coli* using average nucleotide identity (ANI) (ANI > 94.00%) (Fig. 2), which was consistent with the above-identified results using MALDI-TOF MS. In addition, there was a close phylogenetic relationship among 114 isolates based on SNP analysis (Fig. S1). In terms of time dimension, the number of SNPs between two isolates found in different years could be less than 20, such as EC693A1 and EC434A2 (Number of SNP = 17). In terms of space dimension, the number of SNPs between two isolates found in different separation sites could also be less than 20, such as EC441B1 from Jinhua and EC618A1 from Quzhou (Number of SNP = 5). To sum up, it was deduced that there might be cross-time and cross-regional transmissions of *E. coli* among pig farms.

### The antimicrobial resistance of 114 *E. coli* isolates

**Fig 3:**
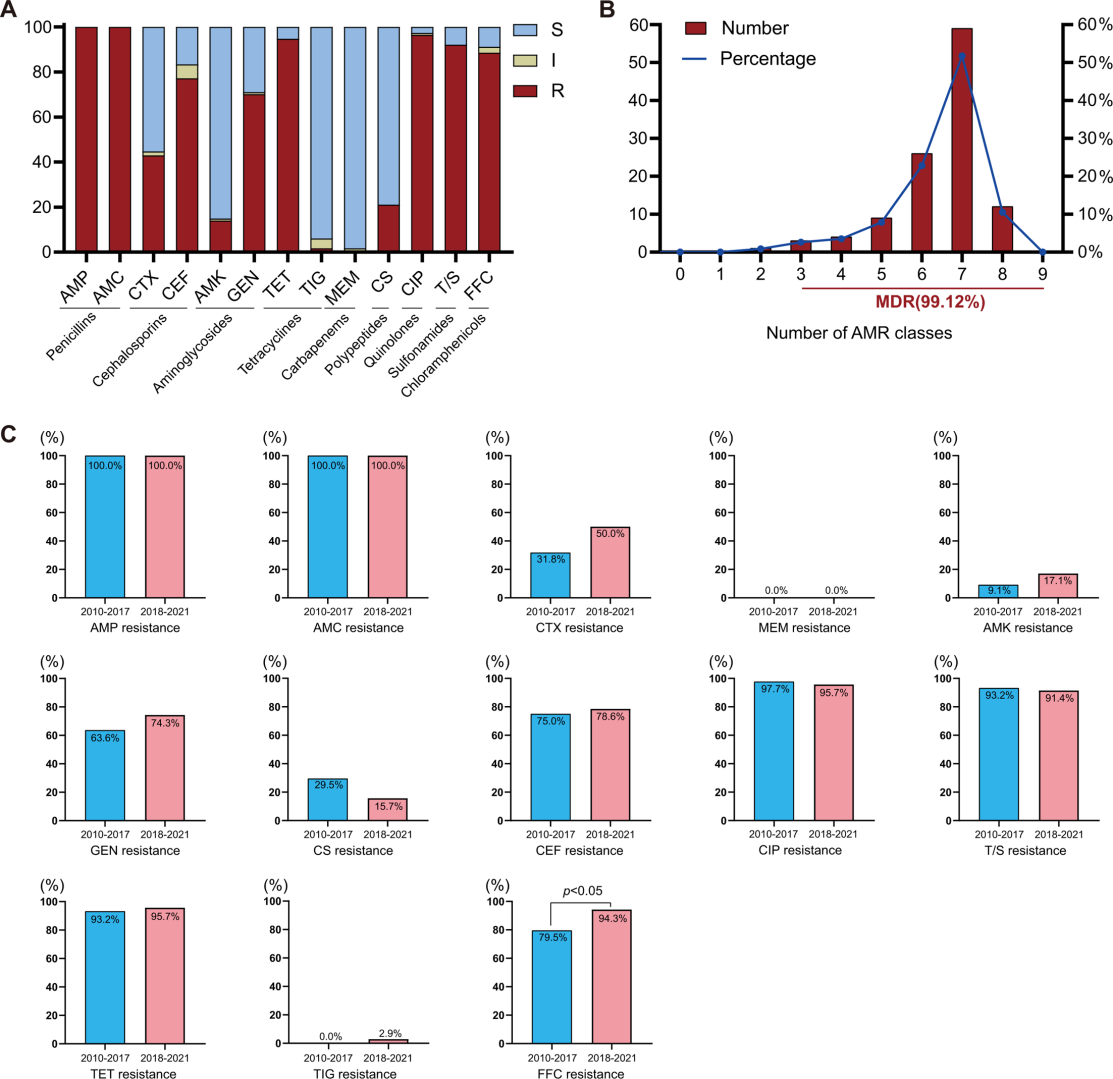
The antimicrobial resistance of 114 *E. coli* isolates. (A) The AMR rates of 114 *E. coli* isolates to 13 antimicrobial agents. (B) The distribution of multidrug-resistant strains. (C) The AMR rates of 44 *E. coli* isolates in 2010–2017 and 70 *E. coli* isolates in 2018–2021 to 13 antibiotics. The Chi-square test was used to analyze the AMR rates in 2010–2017 and 2018–2021.

Among 114 *E. coli* isolates, the highest level of resistance was observed for AMP and AMC, with 100.00% isolates, followed by CIP (96.49%), TET (94.74%), and T/S (92.11%) (Fig. 3A; Fig. S2; Table S2). 21.05% of isolates were resistant to CS, 1.75% of isolates were resistant to TIG, and none of the *E. coli* isolates showed resistance to MEM. A total of 113 *E. coli* isolates (99.12%) were resistant to at least three classes of antimicrobial agents (regarded as multidrug resistant), while 71 isolates (62.28%) showed resistance to seven or more classes of antimicrobial agents (Fig. 3B).

The AMR rates of 44 *E. coli* isolates found in 2010–2017 and 70 *E. coli* isolates found in 2018–2021 were compared. As shown in Fig. 3C, the resistance rate to CS of *E. coli* isolates found in 2018–2021 (15.71%) decreased considerably compared to *E. coli* isolates found in 2010–2017 (29.55%). The resistance rate to FFC of *E. coli* isolates found in 2018–2021 (94.29%) was significantly higher (*P* < 0.05) than *E. coli* isolates found in 2010–2017 (79.55%). While the AMR rates of other antimicrobial agents such as CEF, CIP, and TET showed no significant difference. In particular, the AMR rates of AMP, AMC, MEM, and TIG were not applicable to this analysis.

### Characterization of the AMR-related genome features of *E. coli* isolates

There were 32 different plasmid replicons predicted across all 114 isolates, with an average of 4.9 plasmid replicons in each isolate (Fig. S3). The IncFIB(AP001918) plasmid was predicted in 89 of the 114 isolates (78.07%) and was the most prevalent plasmid type, followed by IncFIC(FII) (44.74%, 51/114) and IncHI2 (36.84%, 42/114).

**Fig 4:**
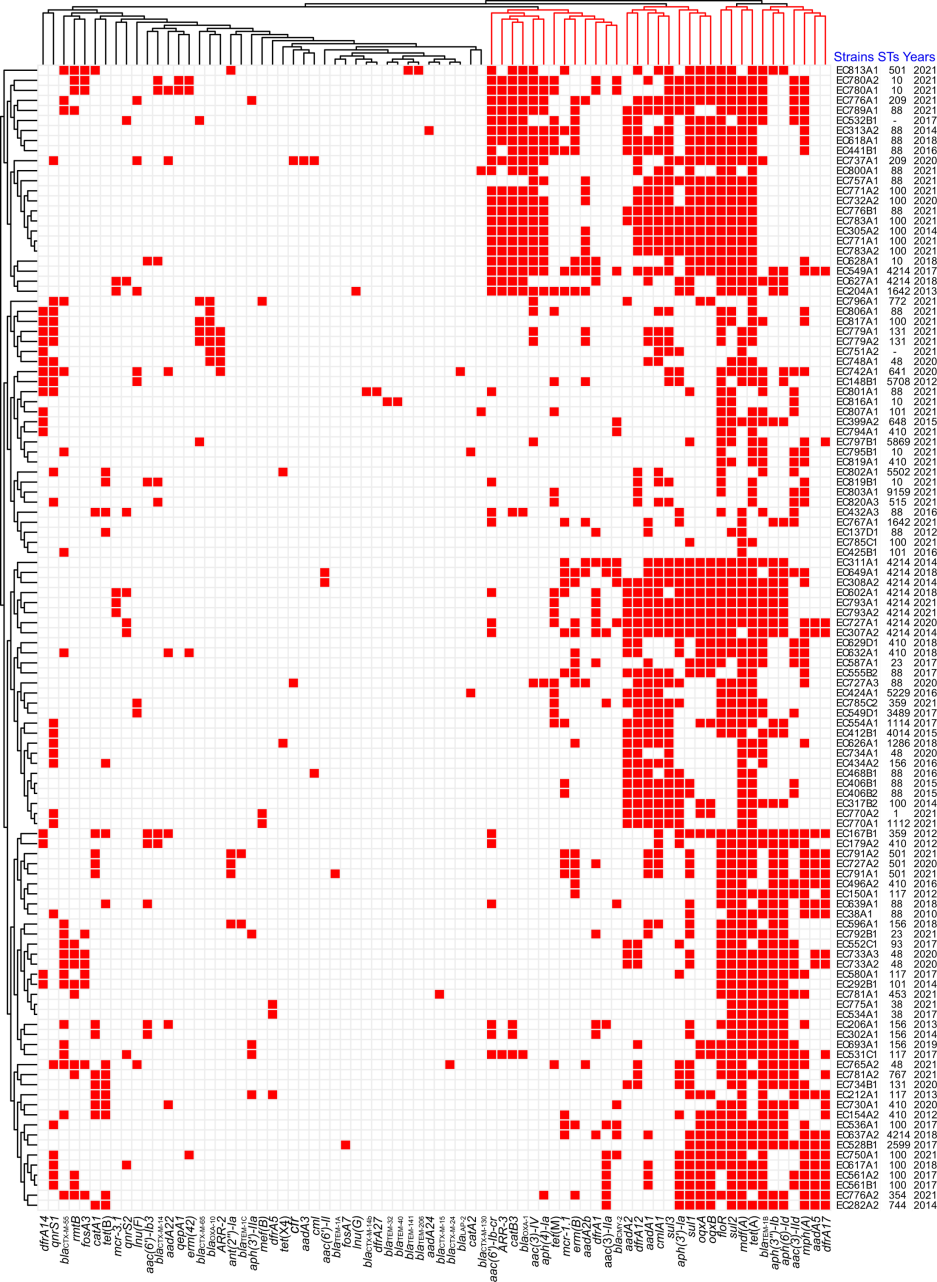
The prediction results of acquired ARGs in 114 *E. coli* isolates.

**Fig 5:**
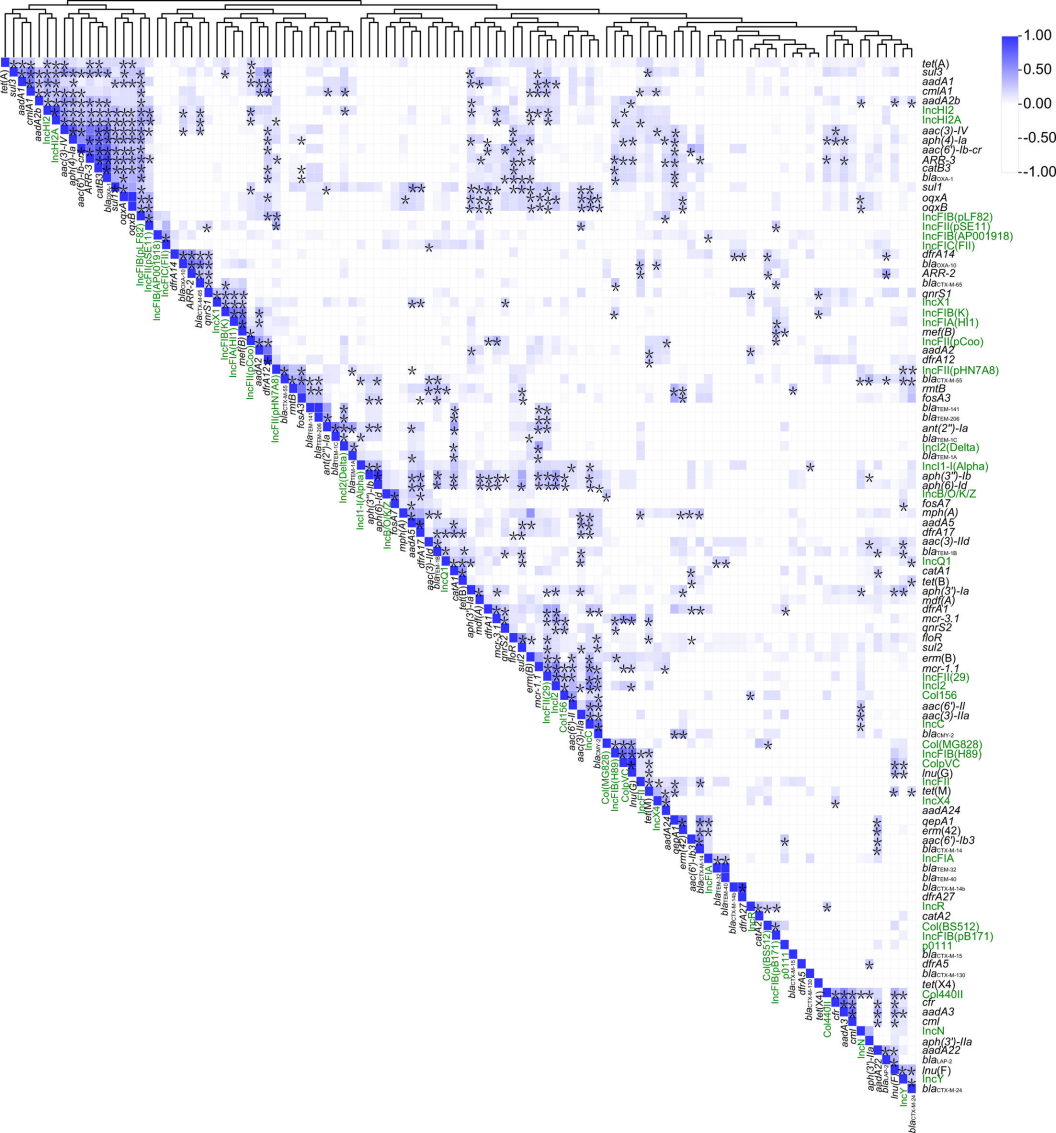
Correlation coefficients for ARGs and plasmid replicons present in 114 *E. coli* isolates. The boxes labeled by “*” indicate a positive correlation with significance calculated at *P* < 0.05. The strength of the blue color in boxes corresponds to the numerical value of the correlation coefficient.

There were 76 ARGs detected in all *E. coli* isolates (Fig. 4), which mainly leads to some resistance phenotypes, including aminoglycoside, amphenicol, beta-lactam, folate pathway antagonist, fosfomycin, lincosamide, macrolide, polymyxin, quinolone, rifamycin, streptogramin A, streptogramin B, and tetracycline. All *E. coli* isolates harbored at least two kinds of ARGs, and 80.70% (92/114) of isolates harbored at least 10 kinds of ARGs. In particular, EC780A1, EC780A2, and EC549A1 isolates were found to harbor 27 kinds of ARGs, which showed serious potential for multidrug resistance. Among all ARGs, the *mdf*(A) gene was mostly detected in 85.09% (97/114) isolates, followed by *tet*(A) (82.46%, 94/114), *floR* (76.32%, 87/114), and *sul2* (75.32%, 87/114). Moreover, there were significant correlations (*P* < 0.05) found for the co-occurrence of several ARGs (Fig. 5), such as *bla*_OXA-1_ with *catB3*, *ARR-3*, or *aac(6′)-Ib-cr*.

Plasmid was usually regarded as the important carrier of ARGs. The physical relationship between plasmid replicons and ARGs was not well detected in the WGS assembly because of the restricted sequencing depth. However, there were significant correlations (*P* < 0.05) found for the co-occurrence of ARGs and plasmid replicons (Fig. 5). For example, the IncHI2 replicon and IncHI2A replicon were strongly correlated with *aadA2b*, *aadA1*, *sul3,* and *tet*(A), and the IncI2 replicon was strongly correlated with *mcr-1*. Genomic analysis of *E. coli* strains EC536A1, EC813A1, EC204A1, and EC602A1 revealed that the *mcr-1* resistance gene was predicted to reside within the IncI2 replicon. Subsequent BLAST analysis against the NCBI database confirmed that the *mcr-1*-containing fragments in 12 additional strains were all mapped to IncI2-type plasmids. Notably, strains EC204A1 and EC602A1 were found to co-harbor both *mcr-1* and *mcr-3* genes, suggesting a potential risk for co-evolution of multidrug resistance. To investigate the horizontal transfer capacity of IncI2 plasmids, conjugation assays were performed on *mcr-1*-bearing IncI2 plasmids. Results demonstrated that 11 out of the tested *mcr-1*-IncI2 plasmids were successfully transferred to the recipient strain *E. coli* strain J53, with conjugation frequencies ranging from 10^−5^ to 10^−2^ (Fig. S4). PCR validation of the transconjugants confirmed the presence of the *uidA* gene, *mcr-1* gene, and IncI2 plasmid replicon sequences (Fig. S4). These findings provide direct experimental evidence that IncI2-type plasmids act as efficient vectors facilitating the horizontal dissemination of the *mcr-1* gene across bacterial strains.

Among the 114 isolates, the *mcr-1* gene was identified in 21 isolates (18.42%, 21/114), while the *mcr-3* gene was found in five isolates (4.39%, 5/114). The designations of these strains were entirely consistent with those of previously reported colistin-resistant strains, and the reverse also held true (Fig. 1B and 4; Table S2). In particular, EC204A1 and EC602A1 isolates harbored both *mcr-1* and *mcr-3*. The detection rate of *mcr-1* in 2018–2021 was significantly lower than in 2010–2017 (Fig. S5), which was consistent with the above comparison analysis results about CS resistance rates in different years. In addition, the PmrB:p.V161G mutation (leading to polymyxin resistance) was detected in 9.65% (11/114) of *E. coli* isolates (data not shown). These 11 isolates included one *mcr*-negative isolate (EC727A), six *mcr-1*-positive isolates (EC307A2, EC308A2, EC311A1, EC549A1, EC637A2, and EC649A1), three *mcr-3*-positive isolates (EC627A1, EC793A1, and EC793A2) and one *mcr-1-mcr-3*-positive isolate (EC602A1). As another notorious ARG, the *tet*(X4) gene was successfully detected in two isolates (EC626A1 and EC802A1). Furthermore, both strains exhibited TIG-resistant phenotypes that were consistent with their corresponding genotypes (Fig. 1B and 4; Table S2).

### Characterization of the virulence-related genome features of *E. coli* isolates

**Fig 6:**
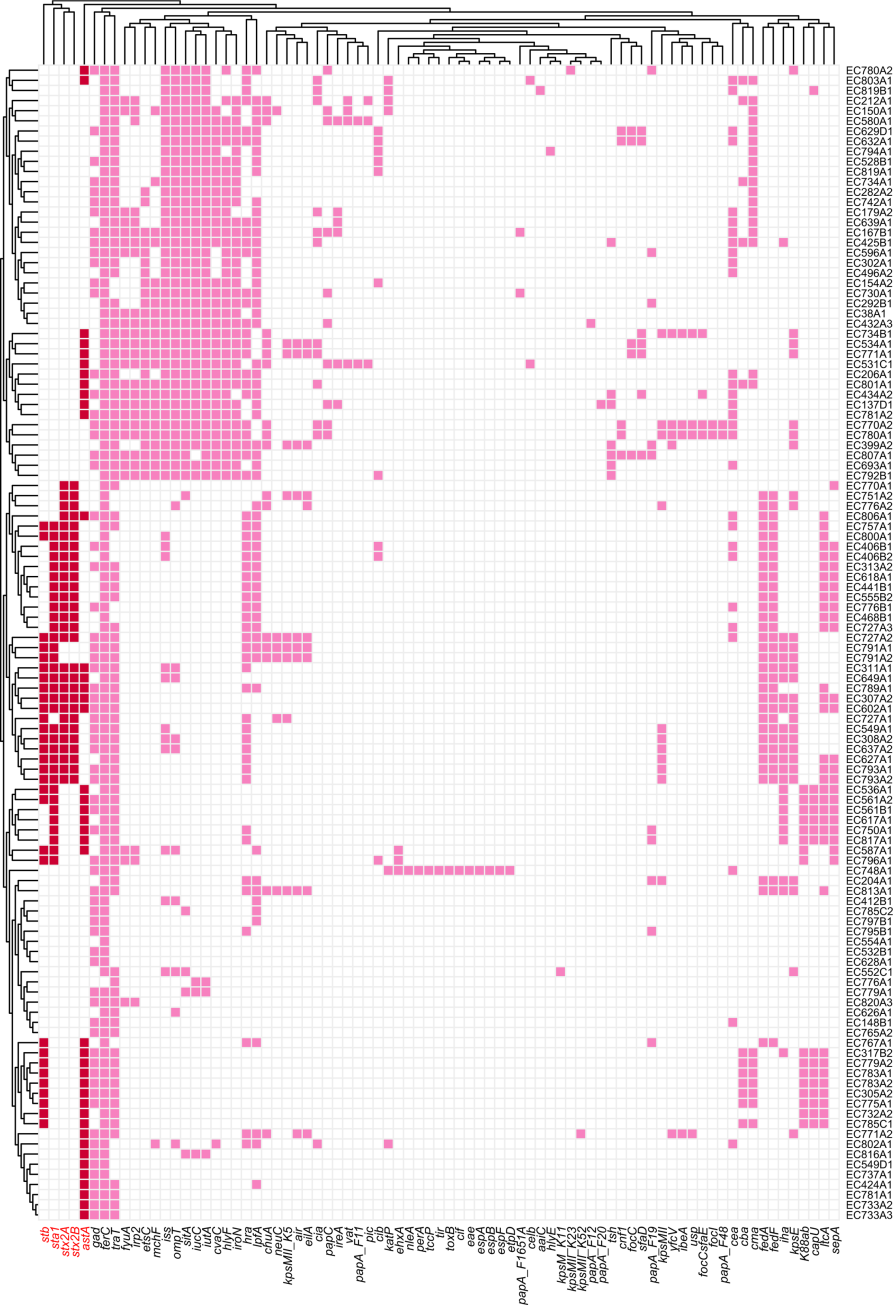
The prediction results of virulence genes in 114 *E. coli* isolates. The red-marked grids represent the well-known high-risk virulence genes of *E. coli*, whereas the pink-marked grids indicate the general virulence genes.

There were 80 virulence genes detected in all 114 isolates (Fig. 6), which mainly lead to some virulence phenotypes including adherence, invasion, iron uptake, secretion system, and toxin. All *E. coli* isolates harbored at least one kind of virulence gene, and 77.19% (88/114) of isolates harbored at least 10 kinds of virulence genes. In particular, EC770A2 and EC780A1 isolates were found to harbor 29 kinds of virulence genes, which showed serious potential for pathogenicity. The *terC* gene was mostly detected in 99.12% (113/114) isolates, followed by *traT* (81.58%, 93/114). In particular, *stb*, *sta1*, *stx2A*, *stx2B,* and *astA* were considered key virulence genes in *E. coli*, and it was observed that 30 (26.32%, 30/114) isolates carried *stb*, 33 (28.95%, 33/114) isolates carried *sta1*, 28 (24.56%, 28/114) isolates carried *stx2A*, 28 (24.56%, 28/114) isolates carried *stx2B*, and 42 (36.84%, 42/114) isolates carried *astA*. A further comparative analysis of the relationship between ST patterns and the above virulence genes (*stb*, *sta1*, *stx2A*, *stx2B,* and *astA*) was performed. As shown in Fig. S6, ST501 was found to have a significant correlation with *stb* and *sta1*, ST100 had a significant correlation with *astA* and *stb*, and both ST88 and ST4214 had significant correlations with *stb, sta1, stx2A,* and *stx2B*. In addition, the positive correlations for the co-occurrence of *astA* and *stb*, and the co-occurrence of *stb*, *sta1*, *stx2A,* and *stx2B* were also found (Fig. S6).

A total of 28 isolates were identified as *stx*-positive and regarded as STEC; a total of 43 isolates were identified as *elt* and/or *estA1*-positive and regarded as ETEC; EC748A1 was identified as *eae*-positive and regarded as EPEC (Table S1). In addition, as serious AMR-*E. coli* isolates, a total of 14 *mcr-1*-positive *E. coli* isolates, two *mcr-3*-positive *E. coli* isolates (including EC627A1 and EC793A2) and one *mcr-1-mcr-3*-co-positive *E. coli* isolate (EC602A1) were identified as ETEC (Table S1). A total of 2 *mcr-1*-positive *E. coli* isolates (including EC307A2 and EC727A2) and one *mcr-3*-positive *E. coli* isolate (EC793A1) were identified as STEC (Table S1). Two *tet*(X4)-positive *E. coli* isolates in this study were not successfully classified.

### Characterization of the genome features of *mcr-1*-harboring and *mcr-3*-harboring plasmids

**Fig 7:**
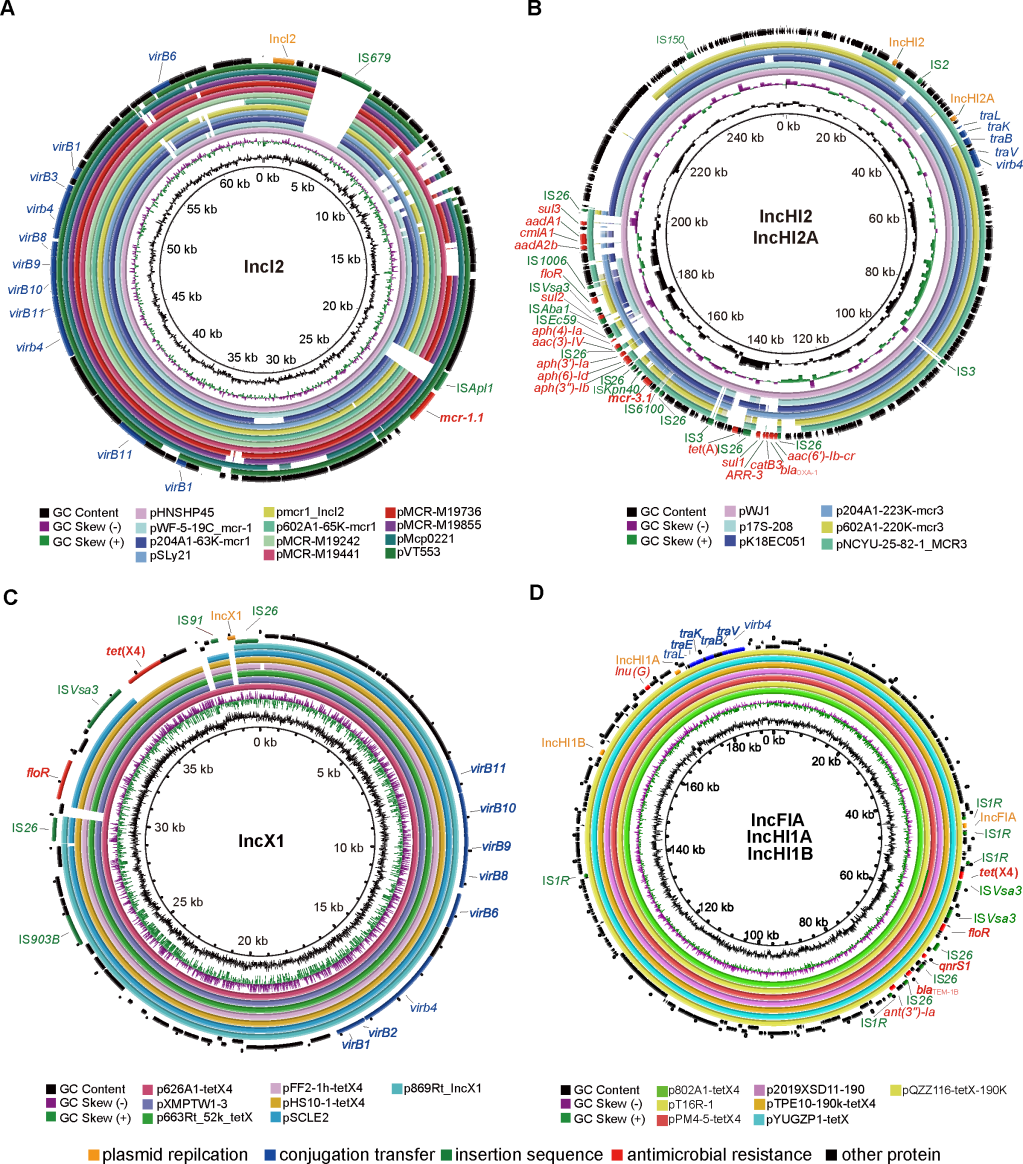
Comparative analysis of mcr-harboring and tet(X4)-harboring plasmids with reported plasmids based on the BLAST Ring Image Generator tool. The genes are color-coded according to functional annotations. (A) Comparative analysis of p204A1-63K-mcr1 and p602A1-65K-mcr1 with reported mcr-1-harboring IncI2 plasmids. (B) Comparative analysis of p204A1-223K-mcr3 and p602A1-220K-mcr3 with reported mcr-3-harboring IncHI2 plasmids. (C) Comparative analysis of p626A1-38K-tetX4 with reported tet(X4)-harboring IncX1 plasmids. (D) Comparative analysis of p802A1-191K-tetX4 with reported tet(X4)-harboring IncFIA-IncHI1A-IncHI1B plasmids.

Based on the above results, 2 *E. coli* isolates (EC204A1 and EC602A1) co-harboring *mcr-1* and *mcr-3* were further chosen to perform complete genome sequencing. Subsequently, *mcr-1*-harboring plasmids were identified in the 2 *E. coli* isolates above, including p204A1-63K-mcr1 (accession no. CP100031) in strain EC204A1, and p602A1-65K-mcr1 (accession no. CP100017) in strain EC602A1 (Fig. 7A). The plasmid p204A1-63K-mcr1, which is an IncI2-type plasmid, was determined to be 63,555 bp in length and have an average GC content of 42.96%. The plasmid p602A1-65K-mcr1, which is also an IncI2-type plasmid, was determined to be 65,277 bp in length and have an average GC content of 44.08%. In particular, both p204A1-63K-mcr1 and p602A1-65K-mcr1 only harbored the *mcr-1* gene. The *mcr-3*-harboring plasmids were also identified in the 2 *E. coli* isolates above, including p204A1-223K-mcr3 (accession no. CP100026) in strain EC204A1, and p602A1-220K-mcr3 (accession no. CP100014) in strain EC602A1 (Fig. 7B). The plasmid p204A1-223K-mcr3, which is a combination of IncHI2- and IncHI2A-type plasmid, was determined to be 223,975 bp in length and have an average GC content of 46.55%. Except for the *mcr-3* gene, 13 ARGs, such as *bla*_OXA-1_, *floR* and *ARR-3*, were also harbored by p204A1-223K-mcr3. The plasmid p602A1-220K-mcr3, which is an IncHI2-type plasmid, was determined to be 220,774 bp in length and have an average GC content of 47.23%. Except for the *mcr-3* gene, 5 ARGs, including *aph(3')-Ia*, *tet*(M), *sul2*, *oqxA,* and *oqxB*, were also harbored by p602A1-220K-mcr3.

The p204A1-63K-mcr1 and p602A1-65K-mcr1 plasmids shared a similar backbone with other *mcr-1*-IncI2 plasmids, such as pHNSHP45, pSLy21, and pMCR-M19242. These plasmids were detected in different kinds of strains and different hosts, suggesting a high prevalence (Fig. 7A). The p204A1-223K-mcr3 and p602A1-220K-mcr3 plasmids were found to have a typical IncHI2 plasmid backbone (Fig. 7B). However, when compared with other *mcr-3*-IncHI2 plasmids (pWJ1, p17S-208, pK18EC051, and pNCYU-25–82-1_MCR3), there were some differences found, mainly including insertion sequences, ARGs, and type IV secretion system (T4SS), which demonstrated a diverse and complex feature of *mcr-3*-IncHI2 plasmids.

**Fig 8:**
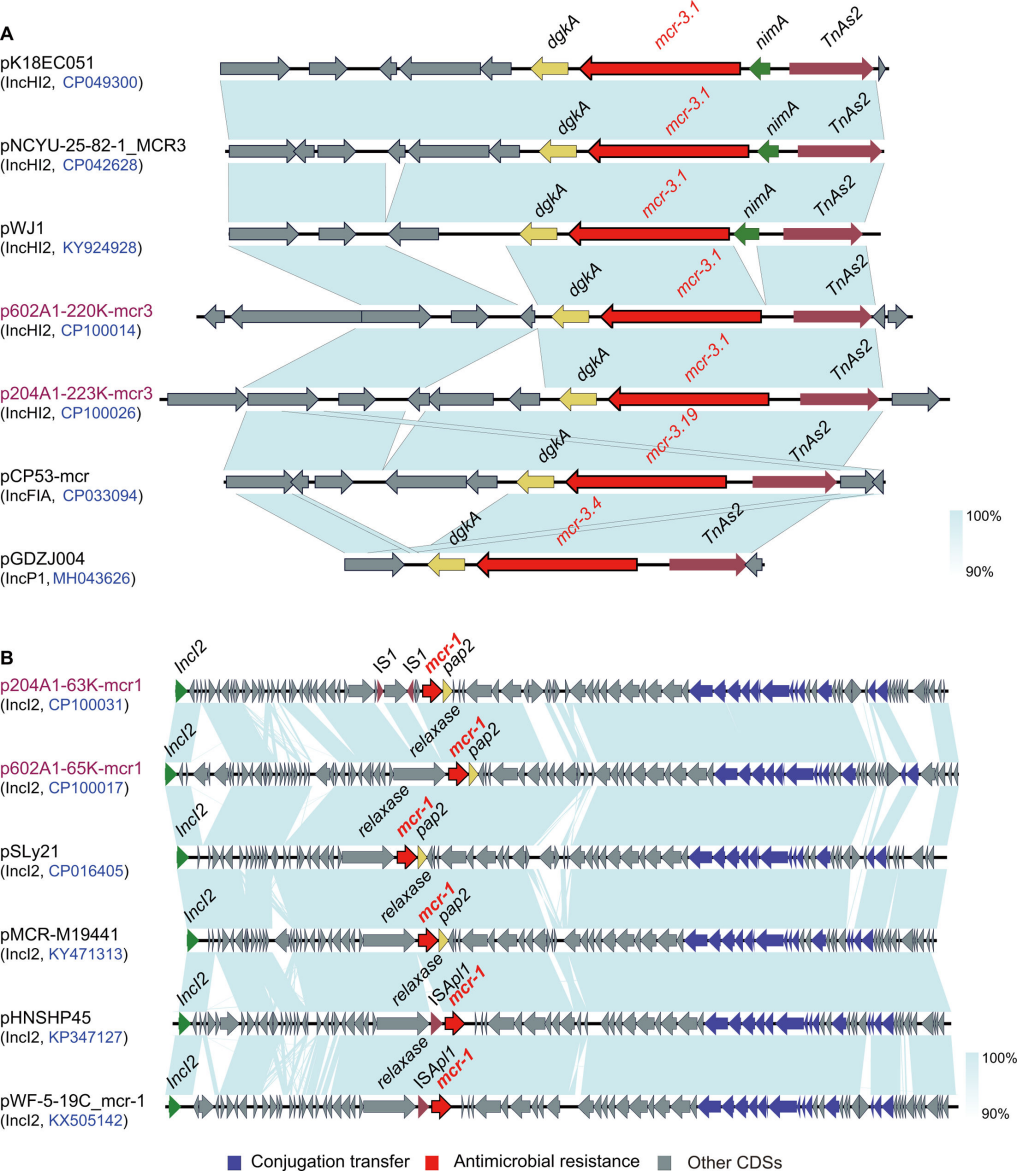
Analysis of the genetic environment of the mcr gene. (A) Genetic environment of mcr-3 in the plasmids p204A1-223K-mcr3 and p602A1-220K-mcr3. (B) Genetic environment of mcr-1 in the plasmids p204A1-63K-mcr1 and p602A1-65K-mcr1.

The genome sequences of plasmids p204A1-63K-mcr1 and p602A1-65K-mcr1 were also used to perform a comparative analysis using Easyfig. As shown in Fig. 8B, plasmid p602A1-65K-mcr1 had a typical structure of *mcr-1-pap2*, which showed a high similarity with the plasmids pSLy21 and pMCR-M19441 and lacked an IS*Apl1* upstream of *mcr-1* compared with plasmids pHNSHP45 and pWF-5–19C_mcr-1. Interestingly, it was found that the relaxase upstream of *mcr-1* was inserted with two IS*1* in the plasmid p204A1-63K-mcr1, and the structure Δrelaxase-IS*1*-Δrelaxase-IS*1-mcr-1-pap2* was obtained, which was a novel genetic feature of the surrounding environment of the *mcr-1* element.

To uncover the genetic context of *mcr-3* in different plasmids in this study, a comparative analysis was performed. As shown in Fig. 8A, some other *mcr-3*-IncHI2 plasmids, such as pK18EC051, pNCYU-25–82-1_MCR3, and pWJ1, had a typical structure of *dgkA-mcr-3-nimA*-transposase. However, a different structure of *dgkA-mcr-3*-transposase was found in p204A1-223K-mcr3 and p602A1-220K-mcr3 plasmids in this study, which lacked a *nimA* gene downstream of *mcr-3*. A further study demonstrated that this *dgkA-mcr-3*-transposase structure could be found in other Inc-type plasmids harboring *mcr-3* variants, such as IncFIA-plasmid pCP53-mcr harboring *mcr-3.19* and IncP1-plasmid pGDZJ004 harboring *mcr-3.4*. To our knowledge, this was the first report of the *dgkA-mcr-3*-transposase structure found in *mcr-3*-IncHI2 plasmid.

### Characterization of the genome features of *tet*(X4)-harboring plasmids

Two *tet*(X4)-harboring plasmids were successfully identified in the two TIG-resistant *E. coli* isolates above, including p626A1-38K-tetX4 (accession no. CP101799) in strain EC626A, and p802A1-191K-tetX4 (accession no. CP101801) in strain EC802A1 (Fig. 7C and D). The plasmid p626A1-38K-tetX4, which is an IncX1-type plasmid, was determined to be 38,924 bp in length and have an average GC content of 44.70% (Fig. 7C). Except for the *tet*(X4) gene, *floR* was also harbored by p626A1-38K-tetX4. The plasmid p802A1-191K-tetX4, which is a combination of IncFIA-, IncHI1A- and IncHI1B-type plasmid, was determined to be 191,488 bp in length and have an average GC content of 46.35% (Fig. 7D). Except for the *tet*(X4) gene, five ARGs (*bla*_TEM-1_, *floR*, *qnrS1*, *aadA22* and *lnu(G*)) were also harbored by p802A1-191K-tetX4.

The p626A1-38K-tetX4 shared a similar backbone with other *tet*(X4)-IncX1 plasmids, such as pFF2-1h-tetX4 and pHS10-1-tetX4, which suggested that IncX-type plasmid could be an important carrier of *tet*(X4) and these plasmids had a highly prevalent presence (Fig. 7C). The p802A1-191K-tetX4 also showed a potential prevalence and shared similar backbone with some other *tet*(X4)-harboring IncFIA-IncHI1A-IncHI1B plasmids. Furthermore, a region including a variety of ARGs and insertion sequences around *tet*(X4) was identified and showed potential for horizontal gene transfer behaviors, which suggested that a long-time gradual evolution of this region might be performed (Fig. 7D).

## DISCUSSION

Swine are recognized as significant hosts of *E. coli*, with pathogenic *E. coli* posing substantial risks to swine health and the productivity of the swine industry (17). Within the “One Health” framework, swine-derived pathogenic *E. coli* is also identified as a major source of clinically relevant *E. coli* infections (18). Identifying the prevalence and characteristics of swine-derived pathogenic *E. coli* is therefore essential not only for advancing the swine industry but also for providing critical data for early warnings of clinical infection risks and public health monitoring (18). In addition, swine-derived pathogenic *E. coli* is considered a potential reservoir and vector for the dissemination of ARGs (18). A thorough understanding of the AMR profiles of swine-derived pathogenic *E. coli* is vital for controlling the evolution and spread of ARGs within the swine-related value chain. In this study, we analyzed 114 *E. coli* isolates from diseased or deceased swine in Zhejiang Province over a 12-year period. Using genomic methods, we investigated the temporal and spatial epidemiological patterns and antimicrobial resistance dynamics of these isolates. These findings provide valuable insights for monitoring *E. coli*-related pig diseases and mitigating public health risks associated with *E. coli*.

In most cases, most *E. coli* could be symbiotic bacteria in swine without causing diseases. Only some hypervirulent clones could result in serious diseases, even death in swine especially piglets (19). Hence, the investigation targeting diseased swine-source *E. coli* could offer some more precise information on *E. coli* that challenges swine health compared with the investigation targeting healthy swine-source *E. coli*. This study is the first to employ genomics to analyze the antimicrobial resistance features of *E. coli* associated with diseased or deceased swine over a large geographic area and an extended time span. The results showed that 99.12% (113/114) of swine disease-related *E. coli* isolates were identified as multidrug-resistant, exceeding the previously reported multidrug resistance rate of 88.68% (1302/1468) for *E. coli* from food animals in developed regions of Eastern China (20) and the 90.54% (1694/1871) rate for *E. coli* from pig farms in mainland China (21). This finding highlights a significant disparity in AMR patterns between *E. coli* isolates from swine clinical cases and those from healthy pigs. Notably, resistance to colistin is particularly alarming, with 21.05% (24/114) of swine disease-related *E. coli* isolates carrying *mcr* genes, in stark contrast to 1.36% (20/1468) and 3.79% (71/1871) reported in the aforementioned surveillance studies (20, 21). To the best of our knowledge, this study is the first to demonstrate that *E. coli* from sick and deceased pigs exhibits significantly more severe AMR than those from healthy food animals. This phenomenon may be attributed to the following factors: *E. coli* associated with host infections and diseases may be influenced by host-specific conditions, displaying an enhanced ability to acquire ARGs, thereby increasing its AMR (22). Furthermore, although the global ban on the use of antibiotics as feed additives has effectively reduced environmental selection pressure, the clinical misuse of antibiotics in veterinary medicine remains prevalent (23, 24). In conclusion, AMR in pathogens linked to zoonotic diseases represents a significant and pressing public health threat. Addressing this issue necessitates comprehensive and rigorous investigations to identify the underlying causes and assess the potential risks associated with high resistance rates. Furthermore, implementing solutions informed by the “One Health” approach is essential to mitigate these challenges effectively.

Previous studies on *E. coli* from sick and deceased pigs have primarily focused on short-term prevalence patterns of individual strains, lacking comprehensive longitudinal epidemiological perspectives. In this study, we examined 12 years of *E. coli* isolates collected from clinical swine in Zhejiang Province. By integrating ST and SNP analyses, we mapped the spatiotemporal transmission patterns and phylogenetic relationships of these strains, creating the first detailed epidemiological framework for swine-associated clinical *E. coli*. The ST distribution demonstrated significant genetic diversity, with dynamic temporal and geographical variations. SNP-based clustering revealed multiple potential clonal transmission events spanning decades and regions, indicating the persistent circulation of high-risk lineages. These findings provide novel insights into the complex dissemination mechanisms driving swine-derived *E. coli* outbreaks and highlight the challenges in controlling their spread. Notably, several dominant STs—including ST38 (25) and ST131 (26)—were identical to pandemic lineages associated with human extraintestinal infections, emphasizing their zoonotic risk. Collectively, this evidence underscores the critical role of clinical swine-derived *E. coli* as One Health hazard and calls for strengthened genomic surveillance systems to mitigate cross-species transmission risks.

This study identified a total of 76 ARGs, highlighting that *E. coli* from diseased pigs could serve as a significant reservoir of ARGs, consistent with the severe multidrug resistance observed earlier. In addition, significant correlations were found among different ARGs and between certain ARGs and plasmids of specific replicon types, suggesting the potential for co-transmission of ARGs via plasmid conjugation. Plasmids are recognized as critical mediators in the dissemination and spread of ARGs (27). The findings further indicate that the high plasticity of plasmid genomic sequences during the long-term evolution of ARGs may promote the frequent coexistence of multiple ARGs on a single plasmid, thereby facilitating their co-transmission (27). This phenomenon could present a substantial challenge to controlling ARG spread in the era of “restricted antibiotic use.” Consequently, effective control of ARG dissemination may require comprehensive monitoring and prevention strategies on a larger and broader scale to mitigate transmission risks and safeguard public health.

Colistin, a peptide antibiotic, was one of the antibiotics of last resort for the treatment of multidrug-resistant Gram-negative pathogen infections (28). Historically, there has been an increasing trend of colistin-resistant *Enterobacteriaceae* caused by *mcr-1* and its variants, which poses a serious risk to patient health (29). Based on this severe fact, colistin has been banned as a growth-promoting agent in livestock and poultry breeding in China since 2017 (30). Fortunately, both the resistance rates of colistin and the detection rates of *mcr-1* reduced after 2017 in this study, which was consistent with some previous reports (31). This reduction may have been mainly mediated by the following (32): (i) *mcr-1* expression could impose a huge fitness cost for *E. coli* and (ii) the reducing usage of colistin posed a reducing selection pressure for the emergence and development of *mcr-1*. Based on the above results, it was indicated that limiting the usage of colistin was a useful measure to control the development of colistin resistance, which also enlightened us on the control of the resistance of some other antimicrobial agents. This study revealed a significant increase (*P* < 0.01) in the resistance rate of FFC following the implementation of the colistin ban in China in 2017. This trend is likely associated with the substantial rise in FFC usage as a replacement for colistin. The quantity of FFC in China was reported to have significantly increased, reaching 1460.8 tons between 2018 and 2020 (33), potentially driving the enrichment of resistance genes such as *floR* through selection pressure. Co-selection pressure is notably another critical mechanism contributing to the persistent increase in FFC resistance, which is associated with the coexistence of multiple ARGs on a single plasmid, as previously mentioned.

At the same time, serious multidrug resistance (including some highly concerned resistance phenotypes such as CS resistance) in ETEC or STEC was observed. On the one hand, this phenomenon suggested that the AMR of hypervirulent *E. coli* underwent significant development and evolution due to antibiotic selection pressure over a long time, which posed a serious threat to public health. On the other hand, the co-evolution of AMR and hypervirulence in *E. coli* may result in synergistic pathogenesis, where AMR not only facilitates survival during early antibiotic interventions but also prolongs the therapeutic window, allowing hypervirulent clones to establish lethal infections. This serious phenomenon suggested that diseased swine might be a high-risk source of AMR hypervirulent *E. coli*, which posed a serious risk to public health and should be focused on in the daily public health monitoring. Notably, this study presents the first identification of ETEC strains harboring the *mcr-3* gene in China, with some strains simultaneously carrying both the *mcr-1* and *mcr-3* genes. This discovery broadens the understanding of ARG profiles in ETEC. As ETEC is a pathogenic bacterium responsible for severe diarrheal outbreaks in humans and neonatal livestock (34), the combination of its intrinsic virulence factors (e.g., heat-labile and heat-stable enterotoxins) and colistin resistance could render colistin ineffective in managing severe ETEC infections. These findings highlight the significant public health risks posed by colistin-resistant ETEC. Furthermore, the novel detection of ETEC strains carrying *mcr-3*, along with the co-occurrence of *mcr-1* and *mcr-3*, suggests that the dissemination and evolution of *mcr* genes within ETEC are advancing, potentially accelerating the emergence of pan-drug resistance.

In this study, *mcr-1*, *mcr-3,* and *tet*(X4) genes were successfully detected, which indicated that diseased swine-source *E. coli* was an important reservoir of these serious ARGs. In addition, the plasmids harboring *mcr-1* or *tet*(X4) showed a high prevalence, and those similar plasmids were prevalent in different AMR pathogens from different hosts and regions, suggesting that diseased swine-source *E. coli* could be the host of plasmids carrying notorious genes and mediate the development of antibiotic resistance. By contrast, the *mcr-3*-harboring IncHI2 plasmids in this study showed some differences with some other previously reported *mcr-3*-harboring IncHI2 plasmids, which suggested the *mcr-3*-harboring IncHI2 plasmids were undergoing continuous development and evolution. The p204A1-223K-mcr3 plasmid isolated from 2013 in this study emerged earlier than pWJ1, which was first reported as an *mcr-3*-harboring plasmid (35), which suggested that the *mcr-3* gene was prevalent for a longer time than our previous understanding. In addition, when compared with pWJ1, p204A1-223K-mcr3 lacked a series of type IV secretion systems for conjugation transfer, which suggested that improving the ability for conjugation and transfer might be an important development direction of *mcr-3*-harboring IncHI2 plasmids, and long-term monitoring is necessary.

Our study reveals that diseased and deceased swine serve as critical vectors for the dissemination of AMR through high-risk *E. coli* strains over a 12-year period. We identified key resistance determinants—*mcr-1*, *mcr-3*, and *tet*(X4)—that compromise the efficacy of last-resort antibiotics such as colistin. Notably, we report novel transmission patterns, including ETEC strains carrying *mcr-3* and strains harboring both *mcr-1* and *mcr-3*, findings not previously documented. Plasmid-mediated horizontal gene transfer further accelerates AMR spread across species. These results emphasize the urgent need for enhanced surveillance and a comprehensive One Health approach to mitigate escalating AMR risks. Collectively, our findings call for targeted interventions in the swine industry to prevent spillover into human populations. Ultimately, these data provide a critical framework for future AMR control strategies.

## MATERIALS AND METHODS

### Identification of *E. coli* isolates

*E. coli* strains were isolated and identified using the following methods (36): First, the lesions of swine (primarily the spleen, joints, gut, and liver) were suspended in 250 mL of buffered peptone water (BPW, Landbridge, Beijing, China) and incubated at 37°C for 12–18 h. After pre-enrichment, homogenates were streaked onto eosin-methylene blue (EMB) agar plates (Landbridge, Beijing, China) and incubated under identical conditions. A single typical colony from each plate was re-streaked onto fresh EMB agar for purification. Presumptive *E. coli* isolates were confirmed using matrix-assisted laser desorption/ionization time-of-flight mass spectrometry (MALDI-TOF MS; Bruker MALDI Biotyper System, Germany) (37). This method involves irradiating bacterial isolates with a laser to generate protein ion mass spectra, which were compared against the reference database. Identification was validated by high match scores (>2.0) and confidence levels, with *E. coli* ATCC 25922 serving as the quality control strain to ensure procedural accuracy throughout the analysis.

### Antimicrobial susceptibility tests

AMR profiles of recovered *E. coli* isolates were determined by the broth microdilution method (Bio Fosun, Fosun Diagnostics, Shanghai, China) (38). The antibiotic concentration range (μg/mL) of 13 antimicrobial agents used in this assay were as follows: ampicillin (AMP): 2- > 128, amoxicillin and clavulanic acid (AMC): 4/2- > 128/64, cefotaxime (CTX): 0.06- > 8, meropenem (MEM): 0.5- > 16, amikacin (AMK): 2- > 64, gentamicin (GEN): 0.25- > 32, colistin (CS): 0.125- > 8, cephalothin (CEF): 0.25- > 32, ciprofloxacin (CIP): 0.06- > 8, trimethoprim/sulfamethoxazole (T/S): 0.5/9.5- > 16/304, tetracycline (TET): 0.25- > 64, tigecycline (TIG): 0.25- > 32, and florfenicol (FFC): 2- > 128. The breakpoints for each antimicrobial agent were set by the Clinical and Laboratory Standards Institute (CLSI M100-ED31:2021 Performance Standards for Antimicrobial Susceptibility Testing, 31st Edition) (Table S3). *E. coli* ATCC 25922 served as a control strain in all assays.

### Whole-genome sequencing and bioinformatics analysis

Genomic DNA extraction was performed from overnight cultures of isolates grown in Luria-Bertani broth (LB, Landbridge, Beijing, China) at 37°C under 180 rpm shaking conditions using a Bacterial DNA Extraction Kit (Generay Biotech, Shanghai, China) as per the manufacturer’s instructions. Then, the extracted DNA was quantified using the Qubit 2.0 Fluorometer (Invitrogen, USA). All Illumina sequencing libraries were generated using a NEXTflex DNA sequencing kit (Bioo Scientific, USA). The paired-end reads (2 × 150 bp) were checked for quality and trimmed with Trimmomatic v0.36 (39). All low-quality (quality score <20) data were filtered out. All isolates were subjected to WGS using the HiSeq platform (Illumina, USA). The raw sequence reads underwent quality check and were assembled with SPAdes v3.12.0 using “the careful option” (as “—careful” is one of SPAdes’ command line options) (40). QUAST 5.0.2 tool was used to evaluate the quality of the assembled genomes (41). Gene prediction and genome annotation were performed using the NCBI Prokaryotic Genome Annotation Pipeline (42).

Two typical *E. coli* isolates co-harboring *mcr-1* and *mcr-3* and two typical *E. coli* isolates harboring *tet*(X4) in this study were used to perform third-generation WGS using the Nanopore GridlON platform. Genomic DNA was extracted following the previously described method, and libraries were prepared using the SQK-LSK109 kit (Oxford Nanopore Technologies, UK). Base calling and adapter sequence removal were performed using Guppy v3.2.4. Hybrid *de novo* assembly of both short and long reads was conducted using the Unicycler v0.4.4 pipeline (43). Gene prediction and genome annotation were carried out with the NCBI Prokaryotic Genome Annotation Pipeline (42).

The virulence genes and ARGs were predicted by the Abricate 1.0.1 tool, which combines data sets from ResFinder 4.6 (http://genepi.food.dtu.dk/resfinder) with a similarity cut-off of (90% nucleotide identity and 90% minimum coverage), and VFDB database with 70% minimum coverage and 50% nucleotide identity (44, 45). The chromosomal mutations were predicted using PointFinder (44). In addition, plasmid types were detected using the PlasmidFinder 2.1 database (https://cge.food.dtu.dk/services/PlasmidFinder/) with a similarity cut-off of (90% nucleotide identity and 90% minimum coverage) (46). Multilocus sequence typing (MLST) was performed using MLST 2.0 (https://cge.food.dtu.dk/services/MLST/) (47). A minimum spanning tree was generated using GrapeTree software to analyze the distribution of STs of *E. coli* isolates (48). Easyfig (maximum e-value of 0.001 and the identity threshold = 98%) and BRIG (the identity threshold = 50%) were used for comparative analysis of the plasmids (49, 50). The ANI among *E. coli* isolates in this study was analyzed by the software Jspecies, using ncbi-blast-2.2.30 + search under the Java Runtime Environment version 8 (51). For phylogenetic analysis based on the maximum-likelihood (ML) method, the KSNP3 software was utilized (52). Finally, the composition of the phylogenetic tree was refined using MEGA X and iTOL (53, 54).

The identification of extraintestinal pathogenic *E. coli* (ExPEC) and diarrheagenic *E. coli* was performed using ncbi-blast-2.2.30 + search under the Java Runtime Environment version 8. The targets selected for each category were *papA*, *papC*, *sfa*/*foc*, *afa*/*dra*, *kpsMT* II, and *iutA* for ExPEC (55), *eae* for enteropathogenic *E. coli* (EPEC) (56), *stx* for Shiga toxin-producing *E. coli* (STEC) (56), *elt*, *estA1,* and *estA2-4* for enterotoxigenic *E. coli* (ETEC) (57), *ipaH* for enteroinvasive *E. coli* (EIEC) (58), and *aggR* and *aat2* for enteroaggregative *E. coli* (EAEC) (58).

### Conjugation assay

Plasmid conjugation experiments were performed on the *mcr-1*-IncI2-harboring *E. coli* strains as described previously (6). A sodium azide-resistant *E. coli* strain J53 was used as the recipient. The *uidA-*specific primers uidA-F (5′-CGACGGCCTGTGGGCATTCA-3′) and uidA-R (5′-GATCCTCCCTGCTGCGGTTT-3′) for identifying *E. coli*, the *mcr-1-*specific primers mcr-1-F (5′-TGCGCCGATTGGGCTTGATCGTGGC-3′) and mcr-1-R (5′-ATCATAGGCATTGCTGTGCGTCTGC-3′) for detecting the presence of *mcr-1*, the IncI2 plasmid replicon sequence-specific primers IncI2-F (5′-TTGATCGATTGCGCCCATGC-3′) and IncI2-R (5′-TCACAGCAAGCTGCACTTAG-3′) for detecting the presence of IncI2 plasmid, were used to confirm the transconjugants.

### Statistical analysis

The Chi-square test was used to analyze the AMR rates. The heatmaps of the clustering of plasmid replicons, ARGs, and virulence genes were performed by TBtools (59). In this analysis, the presence of the above genes received a score of 1, and the absence received a score of 0. Pearson correlation analysis in GraphPad 8 was used to determine the correlations between STs and virulence genes, and plasmid replicons and ARGs.

## Data Availability

All assembled sequence data in this study are available from BioProject ID: PRJNA827955.

## SUPPLEMENTAL MATERIAL

Supplemental material mbio.00651-25-s0001.docx Fig. S1-S6; Tables S1-S3.

ASM does not own the copyrights to Supplemental Material that may be linked to, or accessed through, an article. The authors have granted ASM a non-exclusive, world-wide license to publish the Supplemental Material files. Please contact the corresponding author directly for reuse.
