# Supplementary material for: Genome-based assessment of antimicrobial resistance of Escherichia coli recovered from diseased swine in eastern China for a 12-year period
Source: mBio. 2025 Apr 17;16(5):e00651-25. doi: 10.1128/mbio.00651-25 (PMC12077178; doi:10.1128/mbio.00651-25)
Supplement: Supplemental material — Fig. S1-S6; Tables S1-S3. [file mbio.00651-25-s0001.docx]

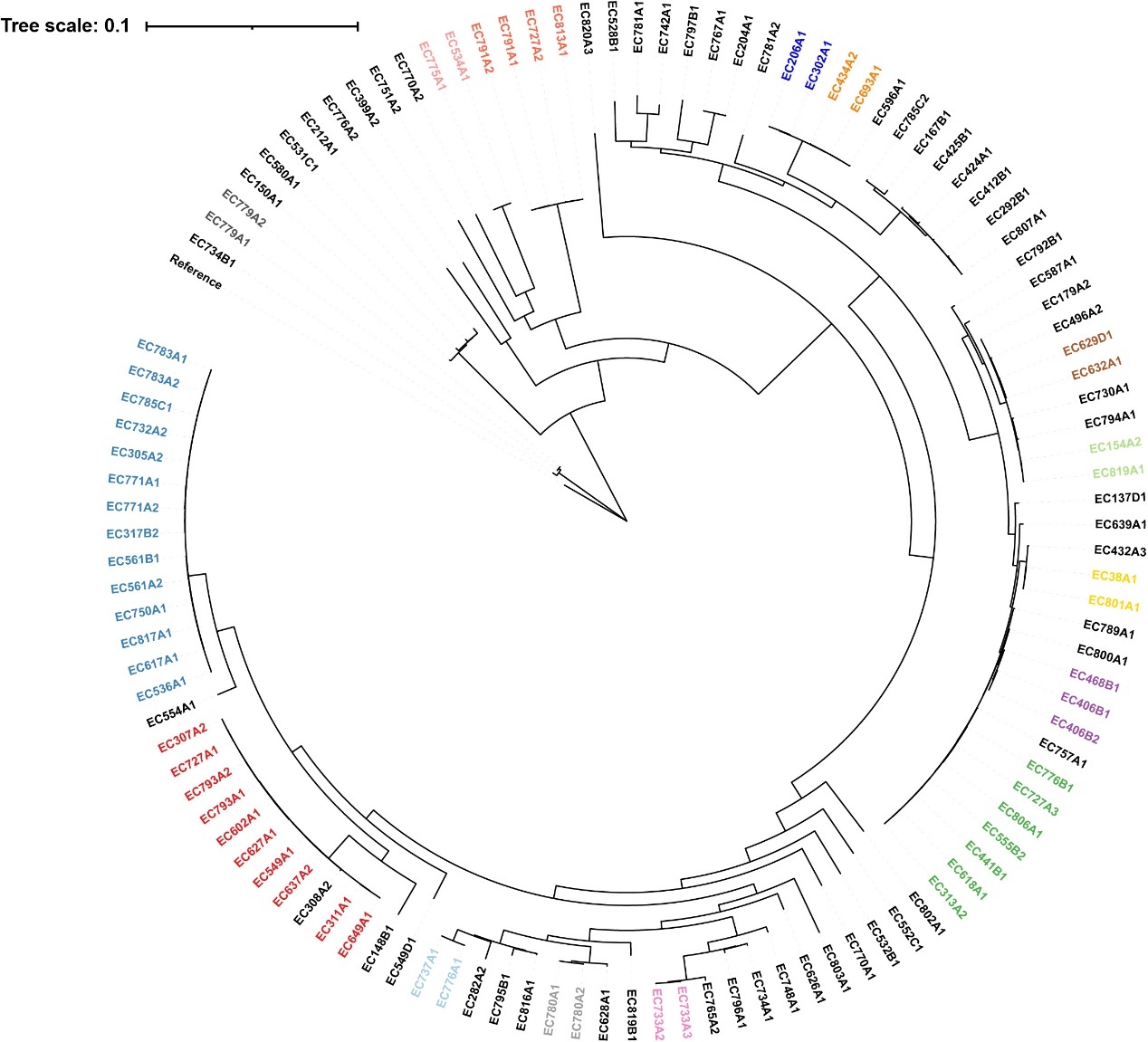


**Fig. S1** Phylogenetic tree of 114 *E. coli* strains. The number of SNP site differences between strains of the same color (excluding black) is fewer than 20.

**
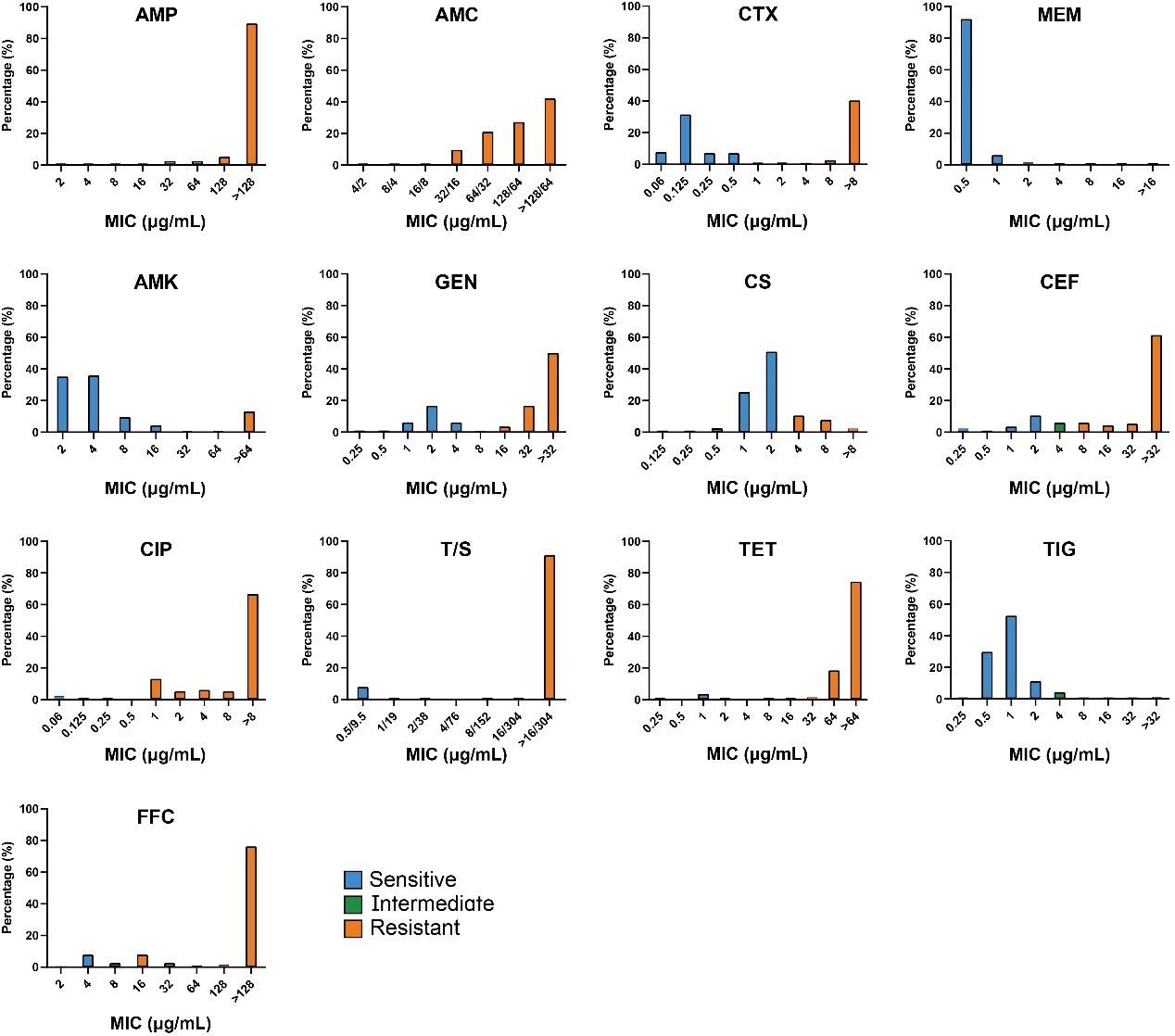
**

**Fig. S2** MIC distributions of 13 antibiotics in isolated 114 *E. coli* strains.

**
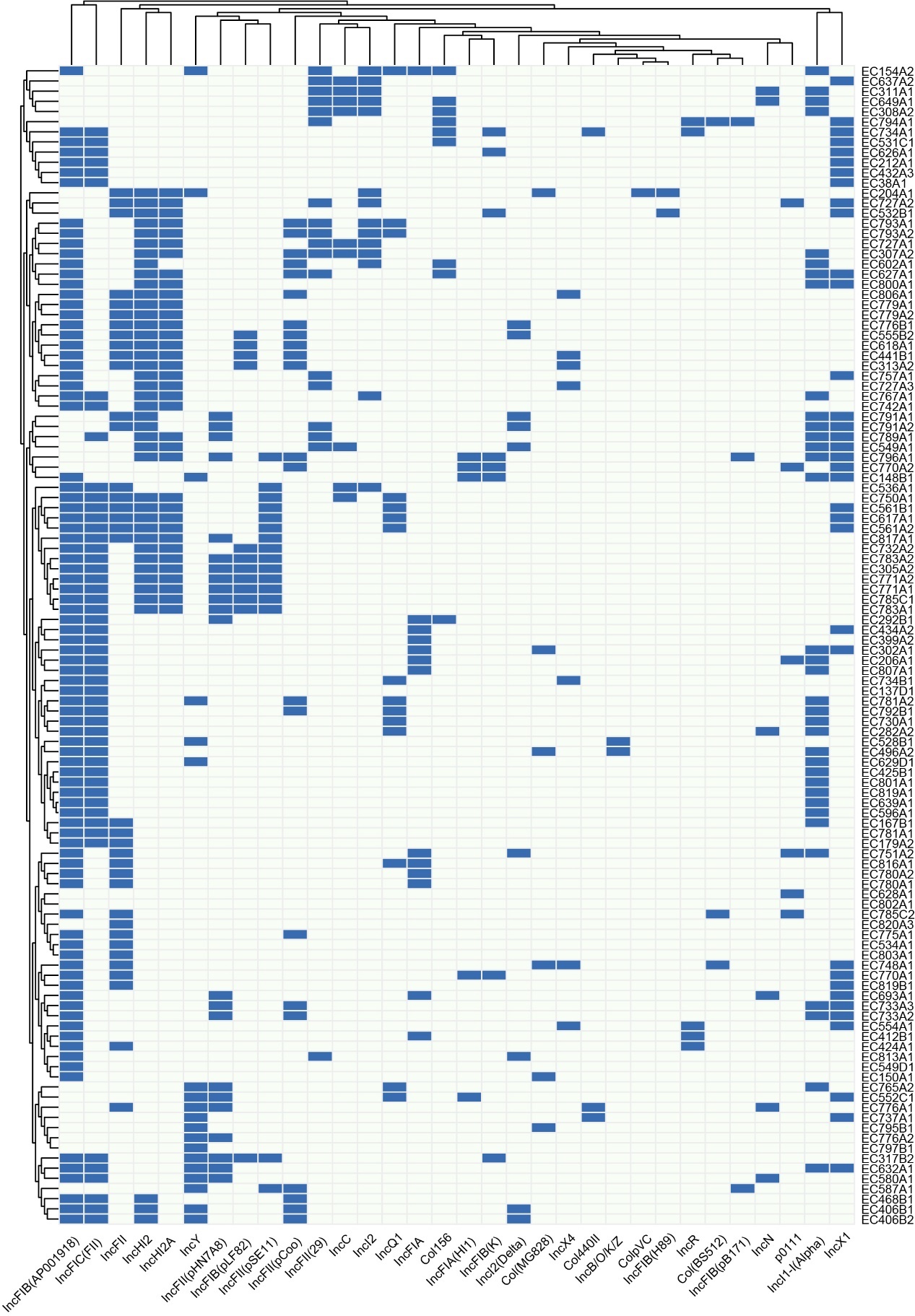
**

**Fig. S3** The types of plasmids carried by 114 *E. coli* isolates


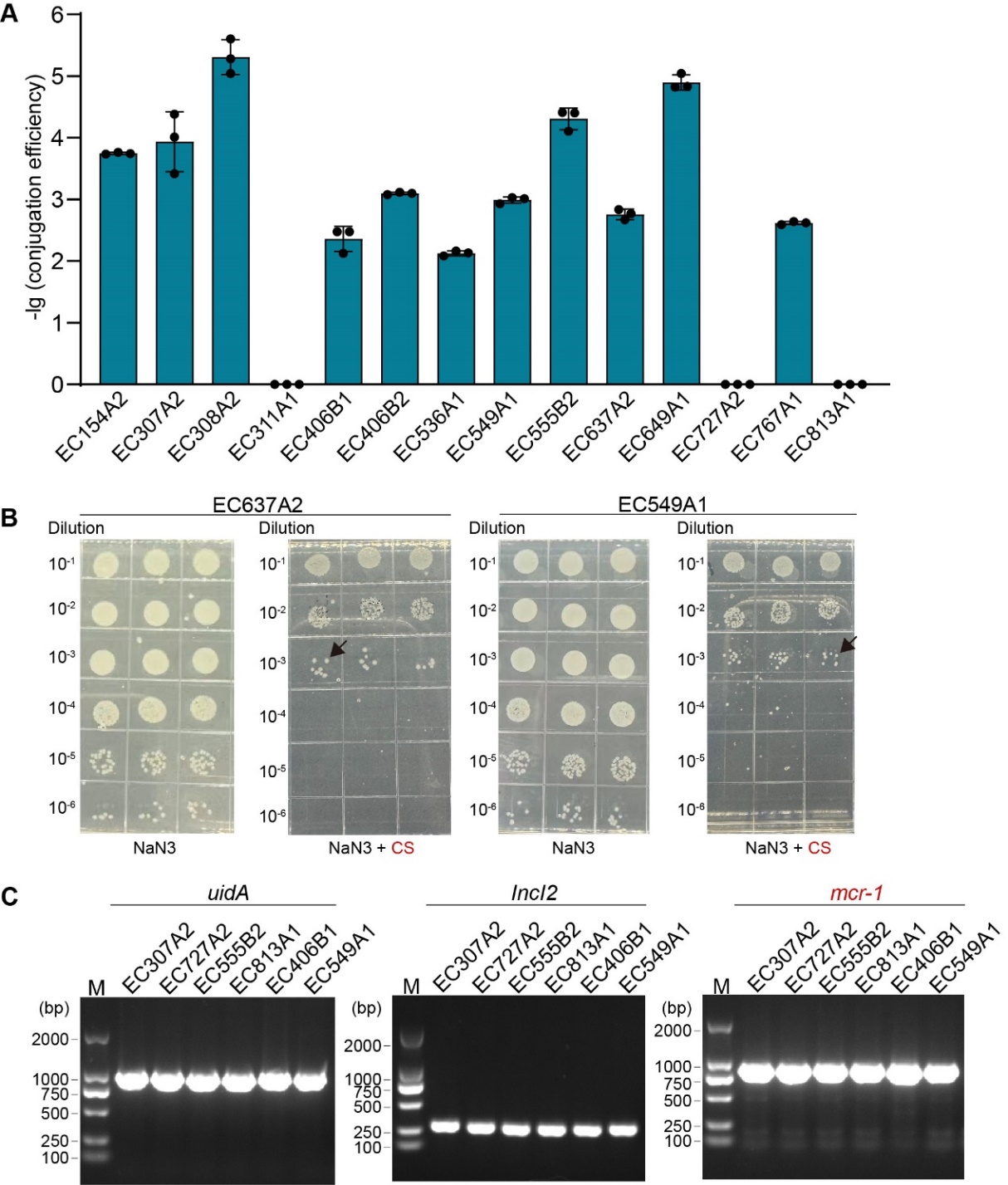


**Fig. S4** The transfer-conjugation test of *mcr-1*-IncI2-harboring *E. coli* isolates. (A) Efficiency of the conjugation transfer test of *mcr-1*-IncI2 plasmids in 14 *E. coli* isolates. (B) Conjugation transfer test of *mcr-1*-IncI2 plasmid-positive *E. coli* strains EC637A2 and EC549A1 with sodium azide-resistant *E. coli* J53 which served as the recipient strain. From left to right are the results under selective pressure of sodium azide, sodium azide and colistin. The arrow represents the transconjugants. (C) PCR verification of transconjugants. The *uidA* specific primers were used for identifying *E. coli*, the *mcr-1* specific primers were used for detecting the presence of *mcr-1*, and the IncI2 plasmid replicon sequence specific primers were used for detecting the presence of IncI2 plasmid.


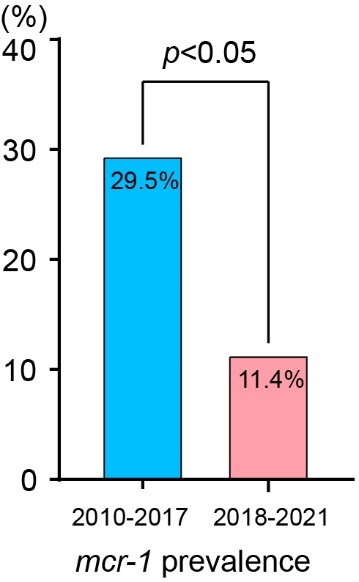


**Fig. S5** The *mcr-1* detection rates of 44 *E. coli* isolates in 2010-2017 and 70 *E. coli* isolates in 2018-2021. The Chi-square test was used to analyze the *mcr-1* detection rates in 2010-2017 and 2018-2021.


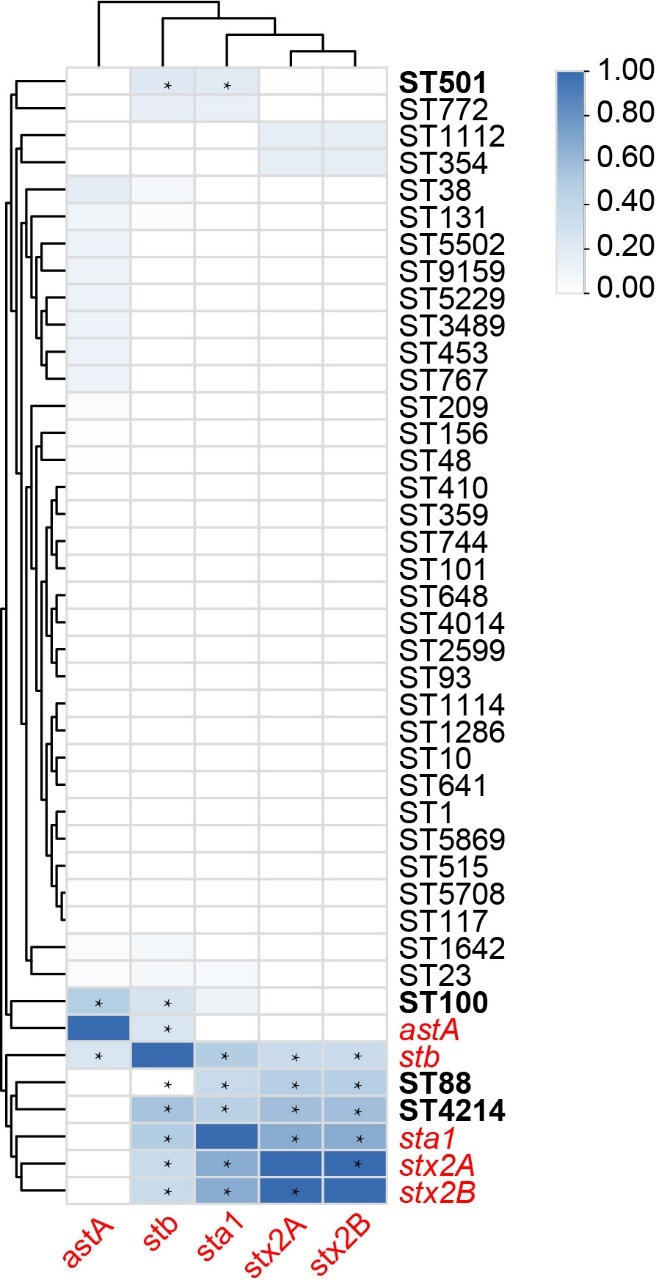


**Fig. S6** Correlation coefficients for STs and virulence genes (*stb*, *sta1*, *stx2A*, *stx2B* and *astA*) present in 114 *E. coli* isolates. The boxes labeled by “*” indicate positive correlation with significance calculated at *p*<0.05. The strength of blue color in boxes corresponds to the numerical value of the correlation coefficient.

**Table S1** The information of *Escherichia coli* isolates used in the present study

| **No.** | **Strain** | **Category** | **MLST** | **Organ** | **Location** | **City** | **Date** | **Reported case** | **Host condition** | ***mcr*** | ***tet*(X4)** |
| --- | --- | --- | --- | --- | --- | --- | --- | --- | --- | --- | --- |
| 1 | EC38A1 | - | 88 | spleen | HU1 | Huzhou | 2010.7.30 | Splenomegaly | Death |  |  |
| 2 | EC137D1 | - | 88 | joint | NB1 | Ningbo | 2012.5.24 | Swollen joint | Death |  |  |
| 3 | EC148B1 | - | 5708 | spleen | JX5 | Jiaxing | 2012.8.13 | Splenomegaly | Death |  |  |
| 4 | EC150A1 | - | 117 | spleen | JX5 | Jiaxing | 2012.8.22 | Acute death | Death |  |  |
| 5 | EC154A2 | - | 410 | spleen | QZ6 | Quzhou | 2012.8.31 | Swollen joint | Death | *mcr-1* |  |
| 6 | EC167B1 | - | 359 | spleen | NB1 | Ningbo | 2012.10.16 | Acute death | Death |  |  |
| 7 | EC179A2 | - | 410 | spleen | HA7 | Hangzhou | 2012.12.20 | Splenomegaly | Death |  |  |
| 8 | EC204A1 | - | 1642 | gut | HA6 | Hangzhou | 2013.3.13 | Diarrhea | Death | *mcr-1*,*mcr-3* |  |
| 9 | EC206A1 | - | 156 | liver | QZ2 | Quzhou | 2013.3.21 | Liver abscess | Death |  |  |
| 10 | EC212A1 | - | 117 | joint | SX14 | Shaoxing | 2013.4.1 | Swollen joint | Death |  |  |
| 11 | EC282A2 | - | 744 | bronchus | TZ1 | Taizhou | 2014.3.25 | Pneumonia | Death |  |  |
| 12 | EC292B1 | - | 101 | spleen | JX4 | Jiaxing | 2014.5.26 | Diarrhea | Death |  |  |
| 13 | EC302A1 | - | 156 | spleen | QZ9 | Quzhou | 2014.7.9 | Acute death | Death |  |  |
| 14 | EC305A2 | ETEC | 100 | gut | JH1 | Jinhua | 2014.7.21 | Diarrhea | Death |  |  |
| 15 | EC307A2 | ETEC, STEC | 4214 | gut | QZ3 | Quzhou | 2014.7.25 | Diarrhea | Death | *mcr-1* |  |
| 16 | EC308A2 | ETEC, STEC | 4214 | gut | SX3 | Shaoxing | 2014.8.2 | Diarrhea | Death | *mcr-1* |  |
| 17 | EC311A1 | ETEC, STEC | 4214 | gut | QZ11 | Quzhou | 2014.8.21 | Diarrhea | Death | *mcr-1* |  |
| 18 | EC313A2 | ETEC, STEC | 88 | gut | SX6 | Shaoxing | 2014.8.26 | Diarrhea | Death | *mcr-1* |  |
| 19 | EC317B2 | ETEC | 100 | gut | HU3 | Huzhou | 2014.9.16 | Intestinal ulcer | Death |  |  |
| 20 | EC399A2 | - | 648 | liver | SX7 | Shaoxing | 2015.9.18 | Hepatomegaly | Death |  |  |
| 21 | EC406B1 | ETEC, STEC | 88 | gut | SX4 | Shaoxing | 2015.10.23 | Diarrhea | Death | *mcr-1* |  |
| 22 | EC406B2 | ETEC, STEC | 88 | gut | SX4 | Shaoxing | 2015.10.23 | Diarrhea | Death | *mcr-1* |  |
| 23 | EC412B1 | - | 4014 | gut | HU4 | Huzhou | 2015.11.18 | Diarrhea | Death |  |  |
| 24 | EC424A1 | - | 5229 | heart | JH5 | Jinhua | 2016.1.7 | Intra-abdominal bleeding | Death |  |  |
| 25 | EC425B1 | - | 101 | spleen | QZ8 | Quzhou | 2016.1.9 | Others | Death |  |  |
| 26 | EC432A3 | - | 88 | ascites | HA11 | Hangzhou | 2016.2.19 | Edema disease | Death |  |  |
| 27 | EC434A2 | - | 156 | joint | HA10 | Hangzhou | 2016.2.20 | Others | Death |  |  |
| 28 | EC441B1 | ETEC, STEC | 88 | gut | JH4 | Jinhua | 2016.3.15 | Diarrhea | Death | *mcr-1* |  |
| 29 | EC468B1 | ETEC, STEC | 88 | gut | HU2 | Huzhou | 2016.8.26 | Diarrhea | Death |  |  |
| 30 | EC496A2 | - | 410 | lung | SX11 | Shaoxing | 2016.12.29 | Others | Death |  |  |
| 31 | EC528B1 | - | 2599 | lung | SX9 | Shaoxing | 2017.4.27 | Acute death | Death |  |  |
| 32 | EC531C1 | - | 117 | lung | LS1 | Lishui | 2017.5.19 | Acute death | Death |  |  |
| 33 | EC532B1 | - | Unknown2 | gut | JX4 | Jiaxing | 2017.5.22 | Diarrhea | Death |  |  |
| 34 | EC534A1 | - | 38 | lung | QZ7 | Quzhou | 2017.6.1 | Diarrhea | Death |  |  |
| 35 | EC536A1 | ETEC | 100 | gut | TZ3 | Taizhou | 2017.6.1 | Acute death | Death | *mcr-1* |  |
| 36 | EC549A1 | ETEC, STEC | 4214 | gut | HA9 | Hangzhou | 2017.7.3 | Diarrhea | Death | *mcr-1* |  |
| 37 | EC549D1 | - | 3489 | gut | HA9 | Hangzhou | 2017.7.3 | Diarrhea | Death |  |  |
| 38 | EC552C1 | - | 93 | gut | SX9 | Shaoxing | 2017.7.13 | Diarrhea | Death |  |  |
| 39 | EC555B2 | ETEC, STEC | 88 | gut | WZ1 | Wenzhou | 2017.8.3 | Diarrhea | Death | *mcr-1* |  |
| 40 | EC554A1 | - | 1114 | lung | JX3 | Jiaxing | 2017.8.4 | Pulmonary congestion | Death | *mcr-1* |  |
| 41 | EC561A2 | ETEC | 100 | spleen | LS4 | Lishui | 2017.9.8 | Splenomegaly | Death |  |  |
| 42 | EC561B1 | ETEC | 100 | gut | LS4 | Lishui | 2017.9.8 | Splenomegaly | Death |  |  |
| 43 | EC580A1 | - | 117 | spleen | HA9 | Hangzhou | 2017.12.8 | Splenomegaly | Death |  |  |
| 44 | EC587A1 | ETEC | 23 | feces | JH3 | Jinhua | 2017.12.27 | Diarrhea | Disease |  |  |
| 45 | EC596A1 | - | 156 | lung | JX3 | Jiaxing | 2018.1.9 | Pulmonary congestion | Death |  |  |
| 46 | EC602A1 | ETEC, STEC | 4214 | gut | SX12 | Shaoxing | 2018.3.2 | Diarrhea | Death | *mcr-1*,*mcr-3* |  |
| 47 | EC617A1 | ETEC | 100 | gut | LS3 | Lishui | 2018.5.14 | Acute death | Death |  |  |
| 48 | EC618A1 | ETEC, STEC | 88 | gut | QZ5 | Quzhou | 2018.5.16 | Diarrhea | Death |  |  |
| 49 | EC626A1 | - | 1286 | gut | QZ4 | Quzhou | 2018.6.15 | Pig scour | Death |  | *tet*(X4) |
| 50 | EC627A1 | ETEC, STEC | 4214 | gut | LS2 | Lishui | 2018.6.21 | Diarrhea | Death | *mcr-3* |  |
| 51 | EC628A1 | - | 10 | lung | JH2 | Jinhua | 2018.6.21 | Edema disease | Death |  |  |
| 52 | EC629D1 | - | 410 | gut | JH6 | Jinhua | 2018.6.26 | Others | Death |  |  |
| 53 | EC632A1 | - | 410 | spleen | JH7 | Jinhua | 2018.7.10 | Splenomegaly | Death |  |  |
| 54 | EC637A2 | ETEC, STEC | 4214 | gut | HA8 | Hangzhou | 2018.7.30 | Diarrhea | Death | *mcr-1* |  |
| 55 | EC639A1 | - | 88 | liver | SX1 | Shaoxing | 2018.8.23 | Splenomegaly | Death |  |  |
| 56 | EC649A1 | ETEC, STEC | 4214 | feces | SX2 | Shaoxing | 2018.11.29 | Diarrhea | Disease | *mcr-1* |  |
| 57 | EC693A1 | - | 156 | heart | SX10 | Shaoxing | 2019.8.6 | Diarrhea | Death |  |  |
| 58 | EC727A1 | STEC | 4214 | feces | HU6 | Huzhou | 2020.8.3 | Diarrhea | Disease |  |  |
| 59 | EC727A2 | ETEC, STEC | 501 | feces | HU6 | Huzhou | 2020.8.3 | Diarrhea | Disease | *mcr-1* |  |
| 60 | EC727A3 | ETEC, STEC | 88 | feces | HU6 | Huzhou | 2020.8.3 | Diarrhea | Disease |  |  |
| 61 | EC730A1 | - | 410 | spleen | WZ5 | Wenzhou | 2020.9.1 | Splenomegaly | Death |  |  |
| 62 | EC733A2 | - | 48 | feces | HA14 | Hangzhou | 2020.9.18 | Others | Disease |  |  |
| 63 | EC733A3 | - | 48 | feces | HA14 | Hangzhou | 2020.9.18 | Others | Disease |  |  |
| 64 | EC732A2 | ETEC | 100 | gut | LS5 | Lishui | 2020.9.21 | Diarrhea | Death |  |  |
| 65 | EC734A1 | - | 48 | heart | SX5 | Shaoxing | 2020.9.23 | Acute death | Death |  |  |
| 66 | EC734B1 | - | 131 | gut | SX5 | Shaoxing | 2020.9.23 | Acute death | Death |  |  |
| 67 | EC737A1 | - | 209 | gut | ZS1 | Zhoushan | 2020.10.10 | Diarrhea | Death |  |  |
| 68 | EC742A1 | - | 641 | chest | HA14 | Hangzhou | 2020.10.29 | Others | Death |  |  |
| 69 | EC748A1 | EPEC | 48 | anal swab | WZ5 | Wenzhou | 2020.11.15 | Diarrhea | Disease |  |  |
| 70 | EC750A1 | ETEC | 100 | gut | HA13 | Hangzhou | 2021.1.6 | Diarrhea | Death |  |  |
| 71 | EC751A2 | STEC | Unknown2 | feces | JX8 | Jiaxing | 2021.1.8 | Diarrhea | Disease |  |  |
| 72 | EC757A1 | ETEC, STEC | 88 | gut | HA12 | Hangzhou | 2021.1.21 | Diarrhea | Death |  |  |
| 73 | EC765A2 | - | 48 | anal swab | WZ5 | Wenzhou | 2021.3.15 | Diarrhea | Disease |  |  |
| 74 | EC767A1 | - | 1642 | gut | NB2 | Ningbo | 2021.3.18 | Diarrhea | Death | *mcr-1* |  |
| 75 | EC770A1 | STEC | 1112 | anal swab | HU5 | Huzhou | 2021.3.28 | Diarrhea | Disease |  |  |
| 76 | EC770A2 | STEC | 1 | anal swab | HU5 | Huzhou | 2021.3.28 | Diarrhea | Disease |  |  |
| 77 | EC771A1 | ETEC | 100 | gut | HA15 | Hangzhou | 2021.3.28 | Diarrhea | Death |  |  |
| 78 | EC771A2 | ETEC | 100 | gut | HA15 | Hangzhou | 2021.3.28 | Diarrhea | Death |  |  |
| 79 | EC775A1 | - | 38 | joint | JX1 | Jiaxing | 2021.4.6 | Swollen joint | Death |  |  |
| 80 | EC776A1 | - | 209 | gut | WZ3 | Wenzhou | 2021.4.7 | Diarrhea | Death |  |  |
| 81 | EC776A2 | - | 354 | gut | WZ3 | Wenzhou | 2021.4.7 | Diarrhea | Death |  |  |
| 82 | EC776B1 | ETEC, STEC | 88 | gut | WZ3 | Wenzhou | 2021.4.7 | Diarrhea | Death |  |  |
| 83 | EC779A1 | - | 131 | feces | QZ12 | Quzhou | 2021.4.15 | Diarrhea | Disease |  |  |
| 84 | EC779A2 | - | 131 | feces | QZ12 | Quzhou | 2021.4.15 | Diarrhea | Disease |  |  |
| 85 | EC780A1 | - | 10 | feces | WZ2 | Wenzhou | 2021.4.21 | Diarrhea | Disease |  |  |
| 86 | EC780A2 | - | 10 | feces | WZ2 | Wenzhou | 2021.4.21 | Diarrhea | Disease |  |  |
| 87 | EC781A1 | - | 453 | anal swab | JX2 | Jiaxing | 2021.4.26 | Diarrhea | Disease |  |  |
| 88 | EC781A2 | - | 767 | anal swab | JX2 | Jiaxing | 2021.4.26 | Diarrhea | Disease |  |  |
| 89 | EC783A1 | ETEC | 100 | gut | TZ4 | Taizhou | 2021.5.12 | Diarrhea | Death |  |  |
| 90 | EC783A2 | ETEC | 100 | gut | TZ4 | Taizhou | 2021.5.12 | Diarrhea | Death |  |  |
| 91 | EC785C1 | ETEC | 100 | gut | SX9 | Shaoxing | 2021.5.17 | Diarrhea | Death |  |  |
| 92 | EC785C2 | - | 359 | heart | SX9 | Shaoxing | 2021.5.17 | Diarrhea | Death |  |  |
| 93 | EC789A1 | ETEC, STEC | 88 | feces | SX16 | Shaoxing | 2021.5.27 | Diarrhea | Disease |  |  |
| 94 | EC791A1 | ETEC | 501 | gut | SX8 | Shaoxing | 2021.6.28 | Diarrhea | Death | *mcr-1* |  |
| 95 | EC791A2 | ETEC | 501 | gut | SX8 | Shaoxing | 2021.6.28 | Diarrhea | Death | *mcr-1* |  |
| 96 | EC792B1 | - | 23 | spleen | LS2 | Lishui | 2021.7.1 | Splenomegaly | Death |  |  |
| 97 | EC793A1 | ETEC, STEC | 4214 | gut | SX15 | Shaoxing | 2021.7.4 | Splenomegaly | Death | *mcr-3* |  |
| 98 | EC793A2 | ETEC, STEC | 4214 | gut | SX15 | Shaoxing | 2021.7.4 | Splenomegaly | Death | *mcr-3* |  |
| 99 | EC794A1 | - | 410 | spleen | QZ1 | Quzhou | 2021.7.17 | Splenomegaly | Death |  |  |
| 100 | EC795B1 | - | 10 | heart | SX13 | Shaoxing | 2021.7.23 | Others | Death |  |  |
| 101 | EC796A1 | ETEC | 772 | gut | WZ4 | Wenzhou | 2021.7.23 | Diarrhea | Death |  |  |
| 102 | EC797B1 | - | 5869 | brain | ZS2 | Zhoushan | 2021.7.29 | Acute death | Death |  |  |
| 103 | EC800A1 | ETEC, STEC | 88 | feces | TZ2 | Taizhou | 2021.8.12 | Diarrhea | Disease |  |  |
| 104 | EC801A1 | - | 88 | anal swab | HA3 | Hangzhou | 2021.8.12 | Diarrhea | Disease |  |  |
| 105 | EC802A1 | - | 5502 | anal swab | HA2 | Hangzhou | 2021.8.12 | Diarrhea | Disease |  | *tet*(X4) |
| 106 | EC803A1 | - | 9159 | anal swab | HA4 | Hangzhou | 2021.8.12 | Diarrhea | Disease |  |  |
| 107 | EC806A1 | ETEC, STEC | 88 | anal swab | HA5 | Hangzhou | 2021.8.12 | Diarrhea | Disease |  |  |
| 108 | EC807A1 | - | 101 | anal swab | WZ5 | Wenzhou | 2021.8.18 | Diarrhea | Disease |  |  |
| 109 | EC813A1 | ETEC | 501 | gut | QZ8 | Quzhou | 2021.9.22 | Gastrointestinal bleeding | Death | *mcr-1* |  |
| 110 | EC816A1 | - | 10 | gut | JX7 | Jiaxing | 2021.9.26 | Diarrhea | Death |  |  |
| 111 | EC817A1 | ETEC | 100 | gut | QZ10 | Quzhou | 2021.9.27 | Diarrhea | Death |  |  |
| 112 | EC819A1 | - | 410 | anal swab | JX6 | Jiaxing | 2021.10.13 | Others | Disease |  |  |
| 113 | EC819B1 | - | 10 | anal swab | JX6 | Jiaxing | 2021.10.13 | Others | Disease |  |  |
| 114 | EC820A3 | - | 515 | anal swab | HA1 | Hangzhou | 2021.10.15 | Others | Disease |  |  |

**Table S2** The MIC values of 13 antimicrobial agents in 114 *E. coli* isolates

| **Strain** | **AMP** | **AMC** | **CTX** | **MEM** | **AMK** | **GEN** | **CS** | **CEF** | **CIP** | **T/S** | **TET** | **TIG** | **FFC** |
| --- | --- | --- | --- | --- | --- | --- | --- | --- | --- | --- | --- | --- | --- |
| EC38A1 | >128 | 64/32 | 0.125 | 0.5 | 2 | 1 | 1 | 2 | 1 | >16/304 | 64 | 1 | 4 |
| EC137D1 | 32 | 32/16 | 0.125 | 0.5 | 4 | 2 | 2 | 32 | >8 | >16/304 | >64 | 1 | 4 |
| EC148B1 | >128 | 64/32 | 0.06 | 0.5 | 2 | 2 | 2 | 2 | 1 | >16/304 | >64 | 1 | 128 |
| EC150A1 | >128 | >128/64 | 0.06 | 0.5 | 2 | >32 | 0.5 | 1 | 1 | >16/304 | >64 | 0.5 | >128 |
| EC154A2 | >128 | >128/64 | >8 | 0.5 | 4 | 4 | 8 | >32 | >8 | >16/304 | >64 | 2 | >128 |
| EC167B1 | 128 | 64/32 | 0.125 | 0.5 | 2 | >32 | 2 | >32 | >8 | >16/304 | >64 | 1 | 4 |
| EC179A2 | >128 | >128/64 | >8 | 0.5 | 4 | >32 | 2 | >32 | >8 | >16/304 | >64 | 2 | >128 |
| EC204A1 | >128 | 128/64 | 0.25 | 0.5 | 8 | 32 | 8 | >32 | >8 | 0.5/9.5 | >64 | 4 | >128 |
| EC206A1 | >128 | 64/32 | >8 | 0.5 | 2 | >32 | 2 | >32 | >8 | >16/304 | 64 | 1 | >128 |
| EC212A1 | >128 | 128/64 | 0.125 | 0.5 | 4 | >32 | 1 | >32 | >8 | >16/304 | >64 | 0.5 | 32 |
| EC282A2 | >128 | >128/64 | 0.125 | 0.5 | 4 | >32 | 1 | 1 | >8 | >16/304 | >64 | 1 | 4 |
| EC292B1 | >128 | >128/64 | >8 | 0.5 | >64 | >32 | 2 | >32 | >8 | >16/304 | 64 | 1 | >128 |
| EC302A1 | >128 | 64/32 | 0.125 | 0.5 | 4 | 4 | 2 | >32 | >8 | >16/304 | >64 | 1 | 8 |
| EC305A2 | >128 | 128/64 | 0.25 | 0.5 | 8 | 32 | 2 | >32 | >8 | >16/304 | >64 | 1 | >128 |
| EC307A2 | >128 | 128/64 | 0.06 | 0.5 | 2 | >32 | 8 | >32 | >8 | >16/304 | 64 | 1 | >128 |
| EC308A2 | >128 | >128/64 | >8 | 0.5 | 8 | 4 | 8 | >32 | >8 | >16/304 | >64 | 1 | >128 |
| EC311A1 | >128 | >128/64 | 8 | 0.5 | 2 | 32 | 4 | 4 | 2 | >16/304 | >64 | 1 | >128 |
| EC313A2 | >128 | >128/64 | 0.5 | 0.5 | 8 | >32 | >8 | >32 | >8 | >16/304 | >64 | 0.5 | >128 |
| EC317B2 | 64 | 32/16 | 0.125 | 0.5 | 2 | 1 | 2 | 8 | 4 | >16/304 | >64 | 1 | 32 |
| EC399A2 | >128 | >128/64 | 8 | 0.5 | 4 | 4 | 2 | 2 | 1 | >16/304 | 32 | 0.5 | >128 |
| EC406B1 | 128 | 32/16 | 0.125 | 0.5 | 2 | 32 | 4 | >32 | >8 | >16/304 | >64 | 1 | 4 |
| EC406B2 | >128 | 64/32 | 0.125 | 0.5 | 2 | >32 | 4 | >32 | >8 | >16/304 | >64 | 1 | 8 |
| EC412B1 | >128 | 128/64 | 0.125 | 0.5 | 4 | 2 | 2 | 2 | 1 | >16/304 | >64 | 1 | >128 |
| EC424A1 | 64 | 32/16 | 0.125 | 0.5 | 4 | 4 | 2 | >32 | >8 | >16/304 | >64 | 1 | >128 |
| EC425B1 | >128 | 32/16 | >8 | 0.5 | 4 | 2 | 2 | 32 | >8 | 0.5/9.5 | 1 | 0.5 | 2 |
| EC432A3 | >128 | 128/64 | 0.125 | 0.5 | 4 | 32 | 2 | 16 | >8 | 0.5/9.5 | >64 | 0.5 | >128 |
| EC434A2 | >128 | 64/32 | 0.125 | 0.5 | 2 | 2 | 2 | 2 | 1 | >16/304 | >64 | 0.5 | 16 |
| EC441B1 | >128 | >128/64 | 0.25 | 0.5 | 4 | 32 | >8 | >32 | >8 | >16/304 | >64 | 0.5 | >128 |
| EC468B1 | 128 | 64/32 | 0.125 | 0.5 | 2 | 2 | 2 | >32 | >8 | >16/304 | >64 | 1 | >128 |
| EC496A2 | 128 | 32/16 | 0.125 | 0.5 | 4 | >32 | 2 | >32 | >8 | >16/304 | 1 | 0.5 | >128 |
| EC528B1 | >128 | 64/32 | 0.125 | 0.5 | 2 | >32 | 1 | >32 | >8 | >16/304 | >64 | 2 | >128 |
| EC531C1 | >128 | 128/64 | >8 | 0.5 | 4 | >32 | 2 | >32 | >8 | >16/304 | >64 | 1 | >128 |
| EC532B1 | >128 | >128/64 | >8 | 0.5 | 2 | >32 | 0.5 | >32 | >8 | >16/304 | >64 | 1 | >128 |
| EC534A1 | >128 | 64/32 | 0.125 | 0.5 | 2 | 1 | 2 | 0.25 | 0.06 | >16/304 | >64 | 1 | 8 |
| EC536A1 | >128 | 128/64 | >8 | 0.5 | 8 | >32 | 4 | >32 | >8 | >16/304 | >64 | 1 | >128 |
| EC549A1 | >128 | >128/64 | >8 | 0.5 | 8 | >32 | 4 | >32 | >8 | >16/304 | >64 | 0.5 | >128 |
| EC549D1 | >128 | >128/64 | 0.125 | 1 | 4 | >32 | 2 | 1 | 1 | >16/304 | >64 | 1 | >128 |
| EC552C1 | >128 | 128/64 | >8 | 0.5 | >64 | >32 | 2 | >32 | >8 | >16/304 | 0.5 | 0.5 | >128 |
| EC555B2 | >128 | >128/64 | 2 | 0.5 | 2 | 1 | 4 | >32 | >8 | >16/304 | >64 | 0.5 | 32 |
| EC554A1 | >128 | 64/32 | 0.125 | 0.5 | 2 | 2 | 8 | 16 | 8 | >16/304 | >64 | 1 | >128 |
| EC561A2 | >128 | 64/32 | 0.5 | 0.5 | >64 | >32 | 2 | >32 | >8 | >16/304 | >64 | 2 | 16 |
| EC561B1 | >128 | 64/32 | 0.125 | 0.5 | >64 | >32 | 2 | >32 | >8 | >16/304 | >64 | 2 | 16 |
| EC580A1 | >128 | 64/32 | >8 | 0.5 | 2 | >32 | 1 | 2 | 1 | >16/304 | >64 | 1 | >128 |
| EC587A1 | >128 | 32/16 | 0.06 | 0.5 | 2 | >32 | 1 | 8 | 4 | >16/304 | 64 | 1 | >128 |
| EC596A1 | >128 | 128/64 | >8 | 0.5 | >64 | >32 | 1 | 32 | 8 | >16/304 | 64 | 0.5 | >128 |
| EC602A1 | >128 | 64/32 | 0.06 | 0.5 | 4 | 2 | 4 | >32 | >8 | >16/304 | >64 | 1 | 16 |
| EC617A1 | >128 | 64/32 | 0.125 | 0.5 | 4 | 32 | 2 | >32 | >8 | >16/304 | >64 | 2 | >128 |
| EC618A1 | >128 | >128/64 | >8 | 0.5 | 32 | >32 | 2 | >32 | >8 | >16/304 | >64 | 1 | >128 |
| EC626A1 | >128 | 128/64 | >8 | 0.5 | 4 | >32 | 2 | >32 | >8 | >16/304 | 64 | >32 | >128 |
| EC627A1 | >128 | 64/32 | 0.06 | 1 | 4 | 8 | 4 | 8 | 4 | >16/304 | >64 | 4 | >128 |
| EC628A1 | >128 | 128/64 | 0.125 | 0.5 | 2 | 2 | 2 | >32 | >8 | >16/304 | >64 | 1 | >128 |
| EC629D1 | >128 | 64/32 | 0.125 | 0.5 | 2 | >32 | 1 | >32 | >8 | >16/304 | 64 | 2 | >128 |
| EC632A1 | >128 | 128/64 | >8 | 0.5 | 2 | >32 | 2 | >32 | >8 | >16/304 | >64 | 2 | >128 |
| EC637A2 | >128 | >128/64 | >8 | 0.5 | 4 | 2 | >8 | 16 | 8 | >16/304 | 64 | 1 | >128 |
| EC639A1 | >128 | >128/64 | 8 | 1 | 16 | 4 | 2 | 2 | 1 | >16/304 | >64 | 1 | >128 |
| EC649A1 | >128 | >128/64 | >8 | 0.5 | 16 | >32 | 8 | 4 | 2 | 4/76 | >64 | 0.5 | >128 |
| EC693A1 | >128 | 128/64 | >8 | 0.5 | 2 | >32 | 2 | 2 | 1 | >16/304 | 64 | 1 | >128 |
| EC727A1 | >128 | >128/64 | >8 | 0.5 | 4 | 2 | 2 | >32 | >8 | >16/304 | >64 | 0.5 | >128 |
| EC727A2 | >128 | 64/32 | 0.125 | 0.5 | 4 | 16 | 8 | >32 | >8 | >16/304 | >64 | 1 | >128 |
| EC727A3 | >128 | >128/64 | 0.5 | 0.5 | 2 | 32 | 2 | >32 | >8 | >16/304 | >64 | 2 | >128 |
| EC730A1 | >128 | >128/64 | >8 | 0.5 | 2 | >32 | 1 | >32 | >8 | >16/304 | >64 | 0.5 | >128 |
| EC733A2 | >128 | >128/64 | >8 | 0.5 | >64 | >32 | 2 | >32 | >8 | >16/304 | >64 | 1 | >128 |
| EC733A3 | >128 | >128/64 | >8 | 0.5 | >64 | >32 | 1 | >32 | >8 | >16/304 | >64 | 1 | >128 |
| EC732A2 | >128 | >128/64 | 0.25 | 0.5 | 8 | 32 | 1 | >32 | >8 | >16/304 | 64 | 1 | >128 |
| EC734A1 | >128 | 128/64 | 0.125 | 0.5 | 4 | 2 | 1 | 2 | 1 | >16/304 | >64 | 2 | 4 |
| EC734B1 | >128 | >128/64 | 0.5 | 2 | 4 | >32 | 1 | 2 | 1 | >16/304 | >64 | 1 | 16 |
| EC737A1 | >128 | >128/64 | 0.25 | 0.5 | 4 | 16 | 1 | >32 | >8 | >16/304 | >64 | 1 | >128 |
| EC742A1 | >128 | 128/64 | >8 | 0.5 | 2 | >32 | 2 | 8 | 4 | >16/304 | >64 | 4 | >128 |
| EC748A1 | >128 | >128/64 | 0.125 | 0.5 | 4 | 2 | 2 | 4 | 1 | >16/304 | >64 | 1 | 4 |
| EC750A1 | >128 | >128/64 | >8 | 0.5 | 2 | >32 | 2 | >32 | >8 | >16/304 | 4 | 2 | >128 |
| EC751A2 | >128 | 128/64 | 0.125 | 0.5 | 2 | 2 | 2 | 1 | 0.5 | >16/304 | 1 | 0.5 | 4 |
| EC757A1 | 128 | 64/32 | 0.125 | 1 | 2 | 32 | 2 | >32 | >8 | 0.5/9.5 | >64 | 0.5 | >128 |
| EC765A2 | >128 | >128/64 | >8 | 0.5 | >64 | >32 | 2 | >32 | >8 | >16/304 | >64 | 1 | >128 |
| EC767A1 | >128 | >128/64 | >8 | 1 | 4 | >32 | 8 | >32 | >8 | >16/304 | >64 | 1 | >128 |
| EC770A1 | 64 | 32/16 | 0.125 | 0.5 | 4 | 2 | 1 | 2 | 1 | >16/304 | 64 | 0.5 | 16 |
| EC770A2 | 32 | 32/16 | 0.06 | 0.5 | 2 | 2 | 2 | 4 | 2 | >16/304 | 64 | 0.5 | 16 |
| EC771A1 | >128 | >128/64 | 0.5 | 0.5 | 4 | 32 | 1 | >32 | >8 | >16/304 | >64 | 2 | >128 |
| EC771A2 | >128 | >128/64 | 0.25 | 0.5 | 8 | 32 | 2 | >32 | >8 | >16/304 | >64 | 1 | >128 |
| EC775A1 | >128 | 64/32 | 0.125 | 0.5 | 4 | 1 | 1 | 0.25 | 0.06 | >16/304 | 64 | 1 | 4 |
| EC776A1 | >128 | >128/64 | >8 | 0.5 | >64 | >32 | 1 | >32 | >8 | >16/304 | >64 | 1 | >128 |
| EC776A2 | >128 | 128/64 | >8 | 0.5 | >64 | >32 | 2 | >32 | >8 | >16/304 | >64 | 4 | >128 |
| EC776B1 | >128 | >128/64 | 0.5 | 0.5 | 4 | 32 | 2 | >32 | >8 | >16/304 | >64 | 1 | >128 |
| EC779A1 | >128 | 128/64 | >8 | 0.5 | 2 | 32 | 2 | 16 | 8 | >16/304 | 64 | 1 | >128 |
| EC779A2 | >128 | 128/64 | >8 | 0.5 | 4 | 16 | 2 | 8 | 4 | >16/304 | 64 | 1 | >128 |
| EC780A1 | >128 | >128/64 | >8 | 0.5 | >64 | >32 | 2 | >32 | >8 | >16/304 | >64 | 0.5 | >128 |
| EC780A2 | >128 | >128/64 | >8 | 0.5 | >64 | >32 | 2 | >32 | >8 | >16/304 | >64 | 0.5 | >128 |
| EC781A1 | >128 | 64/32 | >8 | 0.5 | >64 | >32 | 1 | 4 | 2 | 0.5/9.5 | >64 | 1 | >128 |
| EC781A2 | >128 | >128/64 | 2 | 2 | >64 | >32 | 1 | 2 | 1 | >16/304 | >64 | 1 | >128 |
| EC783A1 | >128 | >128/64 | 1 | 0.5 | 16 | >32 | 1 | >32 | >8 | >16/304 | >64 | 1 | >128 |
| EC783A2 | >128 | >128/64 | 1 | 0.5 | 16 | 32 | 1 | >32 | >8 | >16/304 | >64 | 1 | >128 |
| EC785C1 | 128 | 32/16 | 0.125 | 0.5 | 2 | 2 | 2 | 8 | 4 | 0.5/9.5 | 64 | 1 | 16 |
| EC785C2 | >128 | 128/64 | 0.125 | 0.5 | 2 | >32 | 2 | >32 | >8 | >16/304 | >64 | 4 | >128 |
| EC789A1 | >128 | >128/64 | >8 | 0.5 | >64 | >32 | 2 | >32 | >8 | >16/304 | >64 | 1 | >128 |
| EC791A1 | >128 | 64/32 | 0.125 | 0.5 | 2 | 32 | 4 | >32 | >8 | >16/304 | >64 | 0.5 | >128 |
| EC791A2 | >128 | 128/64 | 0.125 | 0.5 | 4 | 32 | 4 | >32 | >8 | >16/304 | >64 | 0.5 | >128 |
| EC792B1 | >128 | 128/64 | >8 | 0.5 | 4 | 2 | 1 | >32 | >8 | >16/304 | >64 | 2 | >128 |
| EC793A1 | >128 | >128/64 | 0.5 | 1 | 2 | 1 | 4 | 4 | 2 | >16/304 | >64 | 1 | >128 |
| EC793A2 | >128 | >128/64 | 0.25 | 0.5 | 2 | 1 | 4 | 4 | 2 | >16/304 | >64 | 0.5 | >128 |
| EC794A1 | >128 | >128/64 | >8 | 0.5 | 2 | 2 | 2 | >32 | >8 | >16/304 | >64 | 0.5 | >128 |
| EC795B1 | >128 | 128/64 | >8 | 0.5 | 4 | >32 | 1 | >32 | >8 | >16/304 | 64 | 1 | >128 |
| EC796A1 | >128 | 128/64 | >8 | 0.5 | 64 | >32 | 1 | 8 | 4 | >16/304 | 64 | 0.5 | >128 |
| EC797B1 | >128 | 128/64 | >8 | 0.5 | 4 | 32 | 2 | >32 | >8 | >16/304 | >64 | 0.5 | >128 |
| EC800A1 | >128 | >128/64 | 0.25 | 0.5 | 4 | >32 | 0.5 | >32 | >8 | >16/304 | 64 | 0.5 | 128 |
| EC801A1 | >128 | 128/64 | >8 | 0.5 | 4 | >32 | 2 | 32 | >8 | >16/304 | 1 | 0.5 | >128 |
| EC802A1 | >128 | 64/32 | 0.06 | 0.5 | 2 | >32 | 1 | >32 | >8 | >16/304 | >64 | >32 | >128 |
| EC803A1 | 32 | 32/16 | 0.06 | 0.5 | 8 | >32 | 2 | 32 | >8 | 0.5/9.5 | >64 | 0.5 | >128 |
| EC806A1 | >128 | >128/64 | 0.5 | 0.5 | 4 | 32 | 1 | >32 | >8 | >16/304 | >64 | 1 | >128 |
| EC807A1 | >128 | 128/64 | >8 | 0.5 | 4 | >32 | 2 | 32 | >8 | >16/304 | 32 | 1 | >128 |
| EC813A1 | >128 | 128/64 | >8 | 0.5 | 8 | >32 | 8 | >32 | >8 | >16/304 | >64 | 0.5 | >128 |
| EC816A1 | >128 | >128/64 | 0.125 | 0.5 | 2 | >32 | 2 | 0.25 | 0.06 | 0.5/9.5 | 64 | 0.5 | 16 |
| EC817A1 | >128 | 128/64 | >8 | 0.5 | 2 | 16 | 2 | >32 | >8 | >16/304 | >64 | 1 | >128 |
| EC819A1 | >128 | 128/64 | 0.125 | 0.5 | 4 | >32 | 1 | >32 | >8 | 0.5/9.5 | >64 | 1 | >128 |
| EC819B1 | >128 | >128/64 | >8 | 1 | 8 | 4 | 1 | >32 | 8 | >16/304 | >64 | 0.5 | >128 |
| EC820A3 | >128 | >128/64 | >8 | 0.5 | 16 | >32 | 2 | 16 | 8 | >16/304 | >64 | 1 | >128 |

**Notes:** The unit of MIC values in the table is µg/mL. AMP: ampicillin; AMC: amoxicillin and clavulanic acid; CTX: cefotaxime; MEM: meropenem; AMK: amikacin; GEN: gentamicin; CS: colistin; CEF: cephalothin; CIP: ciprofloxacin; T/S: trimethoprim/sulfamethoxazole; TET: tetracycline; TIG: tigecycline; FFC: florfenicol.

**Table S3** MIC breakpoints for *E. coli* in this study

| **Antibiotics** | **MIC breakpoints (μg/mL)** | | |
| --- | --- | --- | --- |
|  | **S** | **I** | **R** |
| Ampicillin | ≤8 | 16 | ≥32 |
| Amoxicillin-Clavulanic acid | ≤8/4 | 16/8 | ≥32/16 |
| Cefotaxime | ≤1 | 2 | ≥4 |
| Meropenem | ≤1 | 2 | ≥4 |
| Amikacin | ≤16 | 32 | ≥64 |
| Gentamycin | ≤4 | 8 | ≥16 |
| Colistin | ≤2 | - | ≥4 |
| Cephalothin | ≤2 | 4 | ≥8 |
| Ciprofloxacin | ≤0.25 | 0.5 | ≥1 |
| Trimethoprim-Sulfamethoxazole | ≤2/38 | - | ≥4/76 |
| Tetracycline | ≤4 | 8 | ≥16 |
| Tigecycline | ≤2 | 4 | ≥8 |
| Florfenicol | ≤4 | 8 | ≥16 |

S, susceptible; I, intermediate; R, resistant; “-”, Indicates that there is no corresponding criterion.
